# Breeding Driven Enrichment of Genetic Variation for Key Yield Components and Grain Starch Content Under Drought Stress in Winter Wheat

**DOI:** 10.3389/fpls.2021.684205

**Published:** 2021-08-16

**Authors:** Ahossi Patrice Koua, Benedict Chijioke Oyiga, Mirza Majid Baig, Jens Léon, Agim Ballvora

**Affiliations:** ^1^Department of Plant Breeding, Institut für Nutzpflanzenwissenschaften und Ressourcenschutz (INRES), Rheinische Friedrich-Wilhelms-University, Bonn, Germany; ^2^Field Lab Campus Klein-Altendorf, Rheinische Friedrich-Wilhelms-University, Bonn, Germany

**Keywords:** breeding progress, drought, GWAS, LD block, MTAs, QTL, yield components

## Abstract

Drought is one of the major abiotic stress factors limiting wheat production worldwide, thus threatening food security. The dissection of the genetic footprint of drought stress response offers strong opportunities toward understanding and improving drought tolerance (DT) in wheat. In this study, we investigated the genotypic variability for drought response among 200 diverse wheat cultivars (genotypes) using agronomic, developmental, and grain quality traits (GQT), and conducted genome-wide association studies (GWAS) to uncover the genetic architectures of these important traits. Results indicated significant effects of genotype, water regime and their interactions for all agronomic traits. Grain yield (GY) was the most drought-responsive trait and was highly correlated with kernels number per meter square (KN). Genome-wide association studies revealed 17 and 20 QTL regions under rainfed and drought conditions, respectively, and identified one LD block on chromosome 3A and two others on 5D associated with breeding progress (BP). The major haplotypes of these LD blocks have been positively selected through breeding and are associated with higher starch accumulation and GY under drought conditions. Upon validation, the identified QTL regions caring favorable alleles for high starch and yield will shed light on mechanisms of tolerance to drought and can be used to develop drought resistant cultivars.

## Introduction

Global crop production needs an increase of nearly 70% to meet demand by 2050 (Semenov et al., 2014; Mohammadi, 2018). Wheat is one of the world's most important staple food crops with an annual yield of 765.77 million tonnes in 2019 (FAO, 2021). Wheat plays a major role in global food security. However, its production is highly sensitive to climatic and environmental variations (Porter and Semenov, 2005; Semenov et al., 2014) and to various abiotic stress factors such as drought, excessive water, salinity, cold, etc. It is estimated that abiotic stress can lead to an average yield loss of more than 50% for most major crop plants (Boyer, 1982; Bray, 2000). Drought is one of the major stress factors limiting wheat yield in arid, semi-arid as well as temperate regions around the world (Hoseinlou et al., 2013; Nezhadahmadi et al., 2013). Compared to other natural disasters, drought has the largest spatial extent with nearly 80% of the total cultivated area worldwide (Mohammadi, 2018) and has the longest duration (Sheffield and Wood, 2012).

Drought affects the plant-water relations at all levels from molecular, cellular, and organ, to the whole plant levels (Oyiga et al., 2020). Moreover, drought stress affects plant nutrient uptake, as water is the transport medium from which nutrients are taken up by the plant root systems. Following drought incidence, stomata close progressively with a parallel decline in net photosynthesis owing to metabolic limitations and oxidative damage of chloroplasts (Farooq et al., 2014). The immediate consequence is the production of smaller organs, increased, flower abortion, and reduction in the grain filling period, which subsequently affect crop yield.

Grain filling has a significant effect on final grain production and greatly depends on photosynthesis and redistribution of assimilates from vegetative tissue to the reserve pools. Terminal drought accelerates leaf senescence and reduces photosynthesis (Farooq et al., 2011). Cultivars with the ability to stay green under prolonged drought remain photosynthetically active; thereby possess high spike fertility, which is often highly correlated to the number of kernels per spike (Reynolds et al., 2017; Würschum et al., 2018). Spike fertility and grain filling are major complex traits that could reduce grain yield (GY) by 58–92% under severe drought conditions (Dhanda and Sethi, 2002; Farooq et al., 2014). Modern cultivars had higher spike fertility hence, increased grain number per spikelet than old ones due to their higher assimilates partitioning during pre-flowering periods (Royo et al., 2007). These characteristics are desired and useful in breeding programs (Tshikunde et al., 2019), to improve drought tolerance (DT) in cereals.

Although water deficit stress can occur at any time during the crops growing season, Liu et al. (2005) reported that water deficit at reproductive phase causes the most yield loss. Plants adopt various structural and functional adjustments to overcome the negative effects of water stress, ranging from their phenology, morphology, and anatomical structures to their physiological and biochemical reactions (Fang and Xiong, 2015). These adjustments leading to plant tolerance involve four mechanisms, drought avoidance (DA) (or “shoot dehydration avoidance”), DT, drought escape, and drought recovery (Fang and Xiong, 2015). Drought avoidance and Drought tolerance are the two major mechanisms employed by plants to tolerate mild, moderate, and severe drought (Yue et al., 2006). Drought avoidance refers to morphological change such as leaf rolling, increasing wax accumulation, deep rooting system, and phenological change resulting in reduction or extension of the vegetative stage, while DT is the capability of plants to maintain physiological activities through regulation of genes to reduce or repair damages from drought stress (Yue et al., 2006; Luo, 2010). Presently, irrigation of agricultural areas is also employed to prevent substantial yield reduction imposed by drought. However, it is economically costly for small-scale farmers and a threat to the environment as water from irrigation could arouse land degradation and soil salinization (Stockle, 2001; Muli, 2014). The most relevant and economically sound solution is to breed crops with higher water use efficiency (WUE). Increasing plant water uptake and use efficiency for cultivation in drought-prone environments would require a broad understanding of the morphological, genetic, and physiological mechanisms adopted by plants to cope with water shortage.

The discovery of the genetic basis of GY and its component traits is essential for providing breeders with the tools necessary for the development of drought stress-tolerant cultivars (Kadam et al., 2018). Genetic dissection of complex traits such as GY and related traits has been possible through genome-wide association studies (GWAS) based on linkage disequilibrium (LD) (Contreras-Soto et al., 2017; Fang et al., 2017). Recent technology developments have led not only to the identification of a high number of DNA-markers but also the production of the whole genome sequence draft of several crops including wheat with its large size of ~17 gigabases (Shi and Ling, 2018). Several QTL associated with yield related traits in winter wheat under drought stress conditions have been reported (Xie et al., 2017; Xu et al., 2017; Li et al., 2019). However, to the best of our knowledge this is the unique study done to uncover the genetic architectures of traits that are contributing to improved GY over the wheat breeding history between 1946 and 2013. QTL associated with water stress responses are valuable resources for exploitation in developing drought-tolerant (Farooq et al., 2009, Ashraf, 2010) and high-yielding cultivars. Recent findings suggest that breeding has increased GY through conserving favorable genetic factors and haplotypes involved in stress adaptation (Voss-Fels et al., 2019).

In this research, we used a diversity panel of 200 winter bread wheat cultivars released from 1946 to 2013, and widely used in breeding programs around Germany to screen the genotypic variation in agronomic and grain quality traits (GQT) under different water stress conditions. The main goal was to identify drought-tolerant cultivars and relevant QTL as well as shed some light on the DT mechanisms in wheat. The specific objectives of this study were to: (i) identify agronomic and developmental traits that contribute to enhance GY under drought conditions; (ii) highlight the role played by breeding to enhance GY under drought conditions; (iii) identify QTL region linked to breeding progress (BP) and DT using years of release, agronomic, developmental, and GQT.

## Materials and Methods

### Plant Materials and Growth Conditions

In this study, we tested 200 winter wheat cultivars originating from Europe, mostly Germany, USA, South America, and Asia, and previously described for their productivity under contrasting agrochemical input levels (Voss-Fels et al., 2019). The years of release of cultivars in the core set ranged from 1963 to 2013 including at least three cultivars per decade. These cultivars were assessed under drought and non-drought (control) conditions in 2016–2017 and 2017–2018 growing seasons. The drought stress treatment was under a rainout shelter and the control treatment under rainfed conditions, both at the same location in the experimental station of Campus Klein-Altendorf, University of Bonn (50.61°N, 6.99°E, and 187 m above sea level). The plots under rainout shelter were irrigated by moveable overhead sprinklers set to deliver 36 L/m^2^ water per week at the first 2–4 weeks of the experiment to enable germination and early establishment of the plants. Water stress was introduced by withholding water at BBCH40 [Biologische Bundesanstalt, Bundessortenamt und CHemische Industrie (Lancashire et al., 1991)], corresponding to the pre-booting growth stage and continued until harvesting (BBCH99). The difference of the volumetric content of water between rainfed and drought treatments was around 7% volume of soil, around heading growth stage (Supplementary Figure 1). The soil type of the experimental site is a Haplic Luvisol (World Reference Base for Soil Resources, WRB) derived from loamy silt (Perkons et al., 2014).

The plots were arranged in a randomized sub-block design with three repetitions. To reduce neighbor effects due to considerable differences among the cultivars in plant height (PH) and maturation period, the randomization was done within subgroups according to Voss-Fels et al. (2019). Each plot assigned to one cultivar was a single row of 0.90 m and a space of 0.20 m was kept between rows. Per row were sown 60 seeds per cultivar. The previously tested germination rate was above 95%, and there was no significant variation among cultivars. To avoid border effects and plant damage by the machine while performing regular maintenance, four rows plots were flanked by two border rows. The weather data of the experimental site and the soil moisture content (0–30 cm) and temperature are provided in Supplementary Figure 1.

### Phenotyping of Agronomic, Developmental, and Grain Quality Traits

Agronomic traits included PH, kernels number (KN), and spike number (SN) per meter square, shoot dry matter weight (SDW) which corresponded to the whole plant dry biomass weight (PBW) without GY. Thousand kernel weight (TKW) was estimated as mean value multiplied by 2 after counting three repetitions of 500 seeds using an automatic seed counter. Harvest index (HI) was calculated as the ratio of GY to PBW which included GY. Visual scorings of developmental traits such as plant health state, homogeneity of growth, leaf rolling, and leaf greenness were done according to the methods described by Pask et al. (2012). The developmental growth stages of a core set of 20 cultivars that was selected by principal component analysis (PCA) based on SNP makers to represent the genetic diversity of the wheat panel were visually scored following the BBCH scale to assess the effect of drought on the duration of each stage. The GQT included ratios of grain protein content (GPC), grain starch content (GSC), and the neutral detergent fiber (NDF) measured using near infra-red spectrometry (NIRS) with Diode Array 7250 NIR analyzer (Perten Instruments, Inc., USA, 2021) by following the manufacturer's guidelines. Full description of evaluated traits is provided in Supplementary Table 1.

### Phenotypic Data Analyses

A mixed-linear-model was used to carry out a year-specific analysis of variance (ANOVA) to determine the effects of water regimes, cultivars (genotypes), and their interactions using SAS software (SAS Institute, 2015). Errors due to planting positions (row-and-column effects) in the field plots were corrected by including “Replication/Row^*^Column” (Gilmour et al., 1995): rows crossed with columns nested within replication in the restricted maximum likelihood (REML) approach as random effects; whereas, the genotype and water regime treatment effects were considered to be fixed. Variance component estimation was based on REML (Searle et al., 2009). The best linear unbiased estimates (BLUEs) were computed across each year for each water regime and cultivar according to the model (equation 1) and the resulting values were used in all the subsequent analyses.

(1) $$\begin{array}{r} {\text{P}}_{ijm}=\mu +G_{i}+T_{j}+GT_{ij}+R_{m}+\varepsilon_{ijm} \end{array}$$

where P_*ijm*_ is the response phenotype such as GY of the *i*th genotype, under the *j*th water regime, and the *m*th repetition. μ, the general mean of the study, *G*_*i*_ the fixed effect of the *i*th genotype, *T*_*j*_, the fixed effect of water regime, *GT*_*ij*_, the fixed effect of the *i*th genotype under the *j*th water regime. R_*m*_, the random effect of the *m*th repetition nesting row, column and Row ^*^ Column, while ε_*ijm*_ is the error term.

The variance components due to genotypic ($\sigma_{\text{g}}^{2}$) and water regime ($\sigma_{\text{e}}^{2}$) effects were estimated using a mixed model procedure (SAS Institute, 2015) with both components set as random. The broad-sense heritability (H^2^) for all traits were calculated within each regime using Equation (2) as described by Gitonga et al. (2014), and across water-regimes using Equation (3) described by Piepho and Möhring (2007).

(2) $$\begin{array}{rcl} {\text{H}}^{2} & = & \left(\sigma_{\text{g}}^{2}\right)/\left[\sigma_{\text{g}}^{2}+\sigma_{\text{e}}^{2}/r\right] \end{array}$$

(3) $$\begin{array}{rcl} {\text{H}}^{2} & = & \sigma_{\text{g}}^{2}/\sigma_{\text{p}}^{2}\text{ with }\sigma_{\text{p}}^{2}=\sigma_{\text{g}}^{2}+\sigma_{\text{ge}}^{2}/m+\sigma_{\text{e}}^{2}/rm \end{array}$$

where *r* is the number of replications of each genotype; $\sigma_{\text{p}}^{2}$, the phenotypic variance; $\sigma_{\text{ge}}^{2}$, the variance of genotype^*^water regime interaction, $\sigma_{\text{e}}^{2}$, the residual error variance, and *m*, the number of water regimes.

Pearson correlation analysis of genotypic means was performed to assess the correlation between traits using the package *Performance Analytics* and the PCA was done by *Factominer* and *Factoextra*, both also implemented in R software (R Core Team, 2020). Thereafter, the relationships between GY and traits of interest under each water regime were evaluated with a regression model to quantify the contribution of the trait to GY. The regressions were conducted using lm function in R software.

### Drought Stress Tolerance Estimation and Quantification of the Breeding Progress in Evaluated Traits

The stress weighted performance (SWP) described by Saade et al. (2016) was used to identify the cultivars' DT status using the following formula.

(4) $$\begin{array}{r} \text{SWP}=\text{YS}/\sqrt{\text{YP}} \end{array}$$

where YS and YP are the means values of the trait of interest of the considered cultivar under drought stress and rainfed conditions, respectively. The 200 cultivars were ranked for each trait from the highest down to the lowest trait's SWP values and were classified as drought-tolerant and sensitive according to their overall SWP ranking as described by Oyiga et al. (2016).

The breeding progress was investigated by the absolute (ABP) and the relatives (BPr) indices using a panel of 192 cultivars with known release years. The absolute breeding progress (ABP) (increase per year) was the slope (a) of the linear regression line between the traits of interest against the release years. The relative three decades BP (Lichthardt et al., 2020) was considered as the result of changes in traits performance over years, and was calculated using the formula *BPr* = *(Pi*_2010_ –* Pi*_1980_*)/Pi*_1980_; where *Pi*_2010_ and *Pi*_1980_ were determined using the coefficients obtained with the following equation from the regression model of the ABP.

(5) $$\begin{array}{r} \text{Pi}\left(\text{x}\right)=\text{ax }+\text{b} \end{array}$$

where x corresponds to 2010 or 1980; a is the slope representing the ABP, and b, the intercept. For a trait of interest, we also test the significant difference between the means values of contrasting year of release cultivars groups to confirm the three decades BP in the wheat panel. The group of oldest cultivars were released before 1980 (31 cultivars) and the newest were released after 2010 (30 cultivars).

### Genetic Analysis of the 200 Wheat Diversity Panel

We used for the genetic analysis, a set of 24,216 SNP markers evenly covering all 21 chromosomes of wheat as described by Dadshani et al. (2021). Detailed information of SNP genotyping, population structure (PS), LD analyses of the diversity panel, and the marker–trait association tests through GWAS have been described in Koua et al. (Unpublished data). Briefly, the structure in the wheat panel was analyzed using PS of the wheat panel was inferred using the model-based clustering method implemented in STRUCTURE software (Pritchard and Przeworski, 2001; Falush et al., 2007), along with the delta K approach to identify the true K (Evanno et al., 2005).

The GWAS was performed with two software programs: TASSEL 5.2.13 (Bradbury et al., 2007) and *rrBLUP* package in R (R Core Team, 2020). Both GWAS were conducted following the model:

(6) $$\begin{array}{r} \text{Y}=\text{X}\alpha +\text{P}\beta +\text{K}\mu +\text{ε} \end{array}$$

where Y is the phenotype of a genotype; α and β are unknown vector containing fixed effects; X the fixed effect of the SNP; P the fixed effect of PS given by PCA matrix that included the first three components; K the random effect of relative kinship among cultivars, and ε the error term, which is assumed to be normally distributed with mean = 0 and variance $\delta_{\text{e}}^{2}$. Both Kinship matrix and PCA matrix were generated in TASSEL. Genome-wide association studies for BP was run with cultivars years of release used as phenotypic values. The congruent significant (*P* <10^−4^) SNP loci identified by both programs were accepted as significant marker-traits associations. Also, FDR correction (Mangiafico, 2015) was applied to accept or reject MTAs with *P* <10^−4^ obtained from only Tassel or rrBLUP. The *P*-value threshold of *P* <10^−4^ to accept significant associations was determined based on the Q–Q plots and distribution of *P*-values.

Detection of significant loci interacting with water regimes through genome-wide locus by water regimes interactions was surveyed using the PROC MIXED procedure in SAS 9.4 (SAS Institute, Cary, NC, USA) which also included the Kinship matrix and PCA matrix from TASSEL. The *P*-value cutoffs for accepting highly significant marker ^*^ treatment interaction associated with a trait were set at 1 × 10^−5^ for PBW, SN, and GY and at 1 × 10^−4^ for kernels number per meter square (KN), kernels number per spikes (KNSp), GPC, and GSC.

### SNP Clustering and Candidate Gene Analysis

The detected marker-trait associations (MTAs) were considered to be in LD if they are located within the interval defined by the chromosomal LD (Breseghello and Sorrells, 2006; Pasam and Sharma, 2014), and were grouped in one SNPs-cluster according to Oyiga et al. (2019). The associated chromosomic regions were further explored using scripts written in R program to identify the probable functionally annotated putative candidate genes (iwgsc_refseqv1.0_ FunctionalAnnotation_v1__HCgenes_v1.0-repr.TEcleaned. TAB). The searches were performed in the genome assembly of *Triticum aestivum* cv. Chinese Spring (IWGSC et al., 2018) and only high confident genes were retained.

## Results

### Agronomic and Grain Quality Traits Were Affected by Drought Stress

A mixed model ANOVA was carried out to estimate the variation components genotype (G), water regime (T). and their interaction effects on evaluated traits (Table 1). In both growing seasons, the agronomic and GQT differed significantly (*P* < 0.001) between water regimes (T) and among genotypes (G) except for SN and NDF in 2018. Genotypes and water regimes were highly interacting in 2017, except for GSC and NDF, meanwhile, in 2018, G ^*^ T interaction effects were highly significant for GY, KNSp, TKW, and GSC. Considering the combined ANOVA of both years, water regimes and genotypes, and their interactions effects were detected for all evaluated traits. Drought caused significant reductions in genotypes performance in most of the traits evaluated, and ranged from 0.11 (NDF) to 79.63% (GY) and from 2.25 (NDF) to 60.42% (GY) in 2017 and 2018, respectively. GY and KN were the most affected traits by drought stress with 68.71 and 66.05% reduction, respectively. Furthermore, drought has significantly decreased the time to reach heading, anthesis, and fruit development growth stages compared to rainfed conditions (Supplementary Table 1B). The coefficients of variation (CV) for all traits were higher under drought compared to rainfed treatment in both years, except for TKW in both years and for PH, NDF in 2017. Broad-sense heritability (H^2^) estimates for some traits such as PBW could differ from control to drought treatment. Interestingly, GY recorded a consistently moderate H^2^ under control and drought conditions. Across both conditions, the higher H^2^ were obtained by PH, TKW, GPC, and GSC in both years. The developmental traits evaluated under drought conditions revealed a highly significant difference among genotypes with high CV of 30.07 and 55.86% in 2017 (Supplementary Table 2), for the relative healthy state (HSr) and relative leaf rolling (LRr), respectively.

**Table 1:** ANOVA and descriptive statistics on agronomic, grain quality traits of 200 wheat genotypes (G) evaluated in two water regimes (T) across 2017 and 2018 years (Y).

**Year**	**Statistics**	**Water Regime**	**Agronomic traits**									**Grain quality**		
			**PH (cm)**	**GY (g/row)**	**SDW (g/row)**	**PBW (g/row)**	**TKW (g)**	**SN**	**KN**	**KNSp**	**HI**	**GPC (%)**	**GSC (%)**	**NDF (%)**
2017	Mean	Rainfed	78.93	203.99	192.42	396.21	39.10	708.16	27900	40.79	0.51	14.47	72.24	18.31
		Drought	56.47	41.51	66.17	109.20	34.74	286.42	6260	21.34	0.37	14.26	71.38	18.29
		Reduction (%)	28.46	79.65	65.61	72.44	11.15	59.55	77.56	47.68	27.21	1.46	1.19	0.11
	CV (%)	Rainfed	10.81	13.30	12.60	11.80	10.07	13.60	14.40	16.49	5.81	5.28	1.64	7.40
		Drought	8.59	31.40	20.70	24.20	9.70	17.23	38.30	24.49	16.38	6.65	1.67	4.81
	Heritability	Rainfed	0.95	0.51	0.75	0.65	0.87	0.68	0.50	0.70	0.42	0.72	0.28	0.33
		Drought	0.41	0.43	0.12	0.24	0.72	0.19	0.57	0.63	0.67	0.51	0.59	0.43
		H^2^	0.62	0.06	0.08	0.10	0.73	0.24	0.10	0.34	0.08	0.52	0.44	0.57
	Treatment effect	T	^***^	^***^	^***^	^***^	^***^	^***^	^***^	^***^	^***^	^**^	^***^	ns
		G	^***^	^***^	^***^	^***^	^***^	^***^	^***^	^***^	^***^	^***^	^***^	^***^
		T^*^G	^***^	^***^	^***^	^***^	^***^	^***^	^***^	^***^	^***^	^*^	ns	ns
2018	Mean	Rainfed	85.44	272.84	302.80	573.44	47.92	771.05	30307	39.98	0.48	14.26	72.91	18.20
		Drought	76.88	107.71	134.05	241.64	42.13	414	13500	32.76	0.44	12.31	73.34	17.79
		Lost (%)	10.02	60.52	55.73	57.86	12.07	46.31	55.45	18.05	7.34	13.63	−0.58	2.25
	CV (%)	Rainfed	10.81	13.30	12.60	11.80	7.52	12.31	15.20	13.73	8.22	5.30	1.39	4.11
		Drought	8.59	31.40	20.70	24.20	6.90	14.46	22.70	17.62	10.53	7.55	1.54	6.56
	Heritability	Rainfed	0.67	0.32	0.06	0.17	0.72	NA	0.27	0.43	0.16	0.59	0.61	0.22
		Drought	0.77	0.46	0.42	0.49	0.46	0.32	0.49	0.43	0.22	0.53	0.55	0.04
		H^2^	0.85	0.33	0.34	0.39	0.68	0.12	0.39	0.22	0.42	0.65	0.64	0.12
	Treatment effect	T	^***^	^***^	^***^	^***^	^***^	^***^	^***^	^***^	^***^	^***^	^***^	^***^
		G	^***^	^***^	^**^	^***^	^***^	ns	^***^	^***^	^***^	^***^	^***^	ns
		T^*^G	ns	^**^	ns	ns	^*^	ns	ns	^***^	ns	ns	^*^	ns
Overall	Mean	Rainfed	82.18	238.415	247.61	484.825	43.51	739.60	29100	40.38	0.50	14.36	72.58	18.25
		Drought	66.67	74.61	100.11	175.42	38.44	350.21	9880	27.05	0.41	13.29	72.36	18.04
		Lost (%)	18.87	68.71	59.57	63.82	11.66	52.65	66.05	33.01	17.62	7.50	0.30	1.18
	Factors effect	Year (Y)	^***^	^***^	^***^	^***^	^***^	^***^	^***^	^***^	^***^	^***^	^***^	^***^
		T	^***^	^***^	^***^	^***^	^***^	^***^	^***^	^***^	^***^	^***^	^**^	^***^
		G	^***^	^***^	^***^	^***^	^***^	^***^	^***^	^***^	^***^	^***^	^***^	^***^
		T^*^G	^*^	^***^	^***^	^***^	^***^	^***^	^***^	^***^	^***^	^***^	^*^	ns
		Y^*^T	^***^	ns	^***^	^***^	^***^	^***^	^***^	^***^	^***^	^***^	^***^	^*^
		Y^*^G	ns	^**^	^***^	^**^	ns	^*^	ns	^**^	^**^	^*^	ns	ns
		Y^*^T^*^G	ns	^**^	ns	^**^	ns	ns	ns	^***^	^***^	ns	ns	ns

*PH, plant height; GY, grain yield; SDW, shoot dry weight; PBW, plant biomass weight; TKW, thousand kernels weight; SN, spike number per meter square; KN, grain number per meter square; KNSp, grain number per spike; HI, harvest index, GPC, grain protein content; GSC, grain starch content; NDF, neutral detergent fiber; CV, coefficient of variation; H^2^, trait heritability estimates.*

*The significance level:*

* *P < 0.05,*

** *P < 0.01,*

*** *P < 0.001; ns = non-significant*.

The genetic relationship among traits under each water regime were evaluated using Pearson correlation coefficients based on cultivar means. Results showed significant (*P* <0.001) correlations among most of the traits under rainfed and water stress in 2017 and 2018 growing seasons (Supplementary Figures 2A,B). The strongest associations were obtained between PBW and GY in 2017 (*r* = 0.91) and 2018 (*r* = 0.84) under rainfed conditions. However, under drought conditions, the highest associations were observed between PBW and GY (*r* = 0.87) in 2017 and between GY and KN (*r* = 0.95) in 2018. Interestingly, the yield component KN recorded the highest and consistent correlation with GY under both water regimes and growing seasons. However, in both planting seasons it was higher under drought compared to rainfed conditions. Among GQT, GSC, and NDF were positively correlated, and both exhibited negative associations with GPC under the two water regimes across growing seasons. For the developmental traits assessed under drought, leaves unrolled state (LRr) were significantly (*P* < 0.001) associated with LGr in both years. Leaves unrolled state recorded the strongest relationship with GY in 2017, while LGr was the most correlated to GY in 2018 (Supplementary Figures 2C,D).

The PCA performed showed the relationship among evaluated traits in growing seasons (Supplementary Figure 3). The first two principal components (PC1 and PC2) explained more than 50% of the total genetic variation under control and drought conditions in 2017 and 2018. The total variance explained by these two components is higher under drought stress when compared to rainfed conditions. The genotypic variation in the PC1 was explained by PBW, GY, and SDW under rainfed conditions in both years, while under drought stress, PC1 was consistently explained by PBW, GY, and KN. The PC2 was explained by GPC, GSC, and PH under drought, whereas under rainfed it was differently explained in both years. Generally, PC1 characterized agronomic traits, while PC2 the GQT (Supplementary Figure 3).

### Contribution of Traits to Grain Yield

The multiple linear regression approaches were exploited to ascertain the relative contribution of each yield component trait to GY. Under rainfed conditions, most agronomic traits such as SN, KN, KNSp, TKW, and SDW contributed to GY in both years except SDW in 2018. However, under drought stress conditions, PH did not affect GY, but KN and TKW had higher effects on GY in 2018 (Supplementary Table 3). Further, simple regression analysis confirmed that the yield components contribution to GY and to its variance differs upon water regimes. The variation in KN, KNSp, and SN significantly explained the variation in GY under drought rather than under rainfed conditions, whereas TKW and SDW explained rather the change in GY under rainfed than under drought conditions (Supplementary Figure 4). The regression GY intercepts under both water regimes were highly different, whereas the slopes under both conditions differed for KN and TKW. The slope of KN was higher under drought compared to the control conditions, while the contrary scheme was observed for TKW (Supplementary Table 5).

### Modern Cultivars Perform Better Under Both Drought Stress and Control Conditions

**Figure 1:**
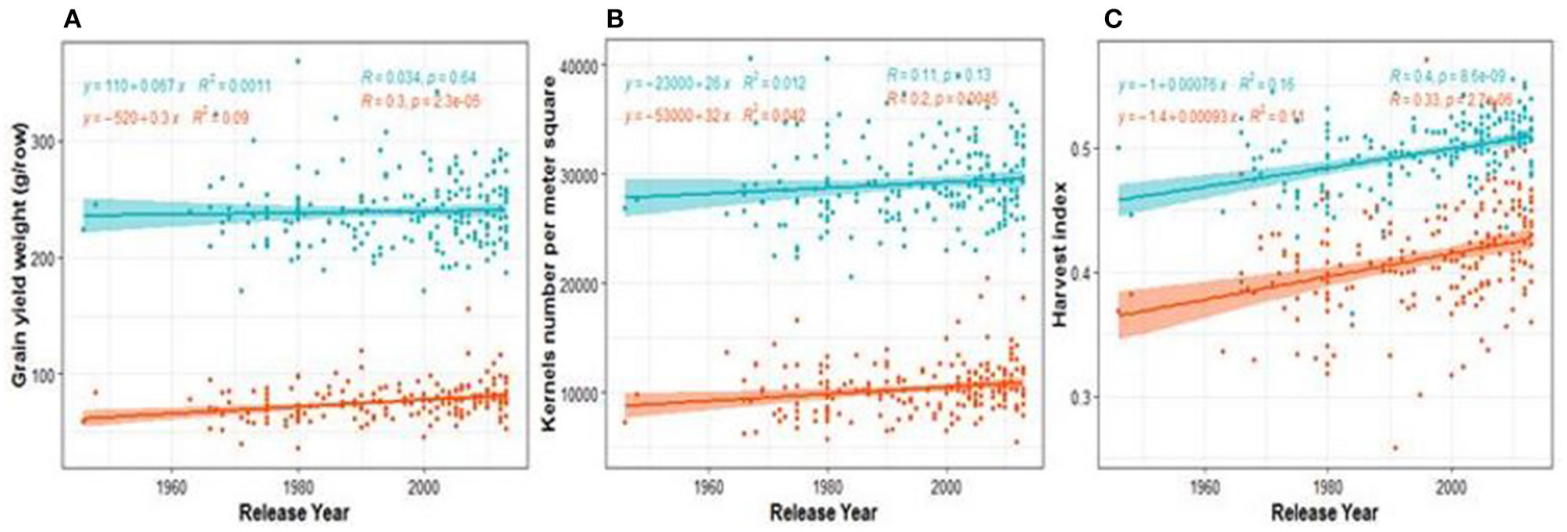
Regression plots showing breeding progress in agronomic traits on Blues values for two growing seasons. Each dot represents a BLUE value of a cultivars and the colored area represents the confidence interval of the regression line. The slopes of the linear regression lines (green lines for rainfed conditions and orange values for droughts stress field) are referred to absolute breeding progress and the relative breeding progress is the ratio between the values in 2010 and 1980 as show in Supplementary Table 4. **(A–C)** are breeding progress in GY, KN, SDW, respectively. The abbreviations of traits names are given in the legend of Table 1.

The ABP in the diversity panel was estimated by testing the significance of the slope (increase per year) from the regression model of the trait of interest against the years of release of cultivars. The results (Figure 1; Supplementary Figure 5) revealed three ABP patterns when the slopes of rainfed and drought treatments are compared (Supplementary Table 4). The first and second patterns were observed when both slopes are either positive or negative, while the third pattern occurs when the slope under drought is opposite sign compared to the one under rainfed (Supplementary Table 4). Although, GY was increasing with year of release in both control and drought conditions, the increase under drought was higher than under rainfed (Figure 1A). We didn't observe any case where breeding increased cultivars performance under rainfed while reducing it under drought. As shown in the scatter plots (Figure 1), the observed variation among cultivars across all regression lines was higher under drought than under control conditions. The relative three decades of breeding progress [BPr (%)] was described by the ratio between the trait value in 2010 and the one in 1980 (Supplementary Table 4). The highest increase was observed for GY and KN with 12.16 and 9.27%, respectively, under drought. Breeding has increased the HI, both under rainfed and drought conditions with a relative increase of 4.52 and 6.32%, respectively. The regressions models of traits vs. year of release comparing the rates of BP under both water regimes showed that the coefficients (intercepts and slopes) observed under drought significantly differed from the ones under rainfed conditions for PBW, SDW, PH, and SN (Supplementary Table 5).

**Figure 2:**
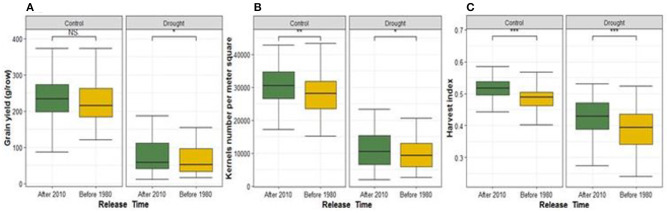
Comparison of breeding progress in agronomic and grain quality traits between two contrasting years of release groups under rainfed (control) and drought stress conditions. The oldest cultivars were released before 1980 (gold color) while the modern were released after 2010 (dark green). **(A–C)** are illustrating the comparison of GY, KN, and HI, respectively. NS means the *P*-value is not significant and *, **, *** mean *P*-value is significant at 0.05, 0.01 and 0.001, respectively.

We compared the performance of the modern cultivars that are the newest (released after 2010) vs. oldest (released before 1980) ones under each water regime using *t*-test of traits mean values between these two contrasting years of release (Figure 2; Supplementary Figure 6). Modern cultivars consistently performed better under both rainfed and drought stress conditions for yield components, GSC and NDF, except for PH and GPC where old cultivars recorded the highest performance (Figure 2; Supplementary Figure 6). Shoot dry matter weight of old cultivars was higher than modern cultivars under rainfed while no significant difference was found under drought stress. Modern cultivars developed more spikes per m^2^ than oldest cultivars under drought stress, whereas under rainfed conditions both groups did not show significant differences.

**Figure 3:**
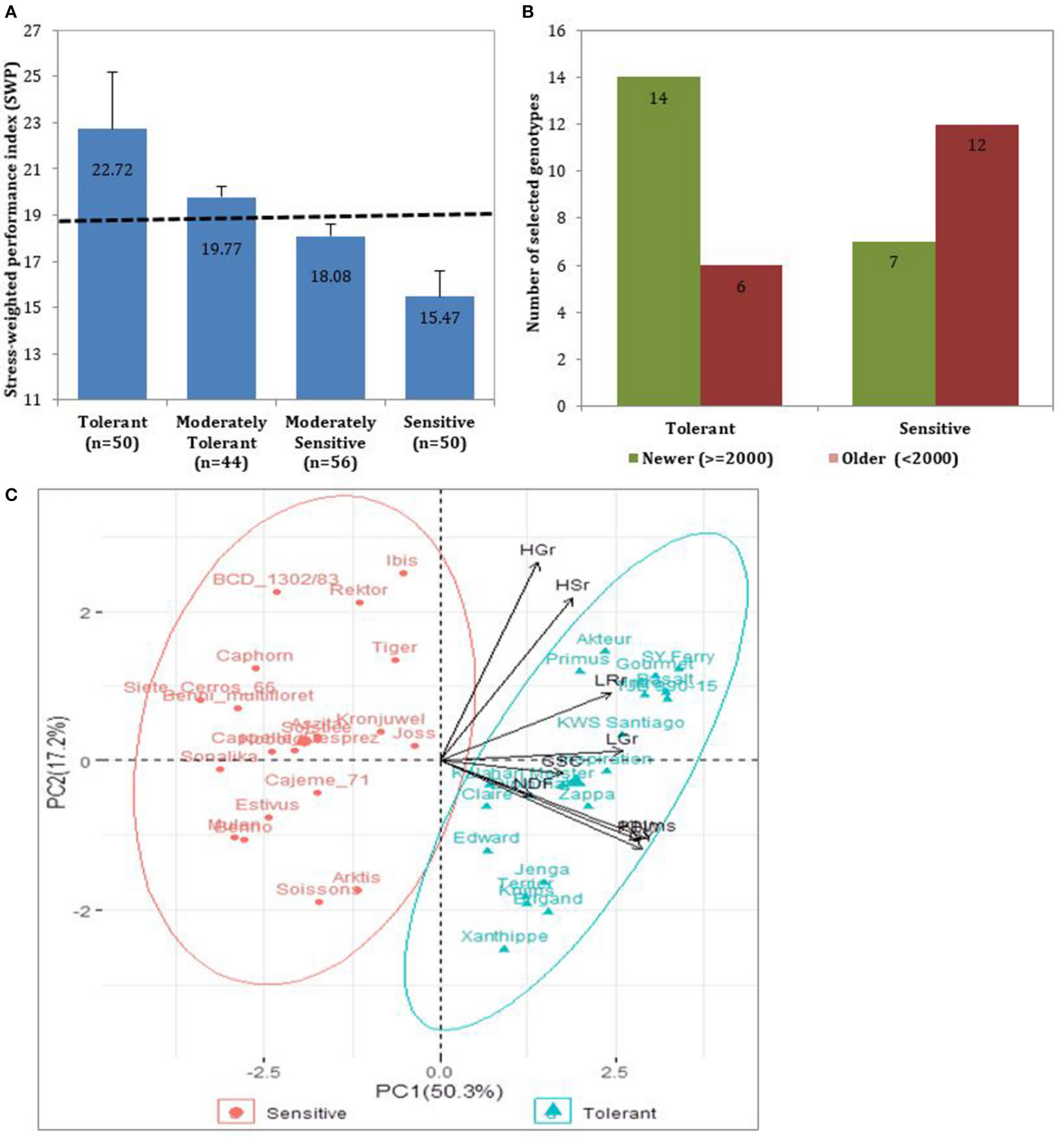
Representation of the studied cultivars based on SWP. **(A)** SWP representation of all the 200 cultivars classified into four drought tolerance groups. The dotted line represents the average SWP value of the entire population (SWP = 18.96). **(B)** The number of selected 20 drought-tolerant and 20 drought-sensitive cultivars. Selected cultivars were classified into Newer (released in/or after 2000) or older (released before 2000). The selection was based on SWP of agronomic and grain quality traits, and the visual scores of developmental traits under drought stress conditions. **(C)** Scatter plot showing clustering of the tolerant (green) and sensitive (red) cultivars based on the PCA analysis of their SWP rankings of evaluated traits.

Further, we calculated the drought stress-weighted performance (SWP) to evaluate the DT status within the evaluated germplasm. Following the SWP index, cultivars with higher SWP values performed better under rainfed conditions and were more drought tolerant than cultivars with smaller values. As shown in Figure 3A, fifty cultivars obtained a SWP above the third quartile (20.62) and were considered drought-tolerant, whereas fifty cultivars with SWP average of 15.75 had their SWP smaller than the first quartile (16.95), hence were considered drought sensitive. The consistently selected tolerant (20) and sensitive (20) from the three categories of traits (agronomic, development, and grain quality), and are presented in Supplementary Table 6. Among them, modern cultivars had the highest SWP indices, indicating they are more tolerant to drought (Figure 3B). The PC1 that explained 50.3% of the total variation in the PCA analysis separated the 20 tolerant and the 20 sensitive cultivars. The parameters that contributed to the difference between cultivars were KN, GY, PBW, LGr, LRr, and GSC with the highest to the lowest in that order (Figure 3C).

### Marker-Trait Associations Detected Under Both Water Regimes and Markers Interacting With Water Regimes

Genome-wide association studies identified 78 significant MTAs (*p* <10^−04^) across 26 QTL regions based on the chromosomal LD (Supplementary Tables xl1, xl2, xl4). In total, 53 MTAs were found under drought and 26 under rainfed conditions. All QTL found under stress conditions are drought responsive since they were not detected under control conditions. The proportion of phenotypic variance explained (PVE) given by all SNP markers averaged 8.27% ranging from 6.84 to 10.27% under rainfed, and averaged 8.26% ranging from 6.12 to 11.29% under drought stress (Supplementary Tables xl1, 7). Chromosomes 7B, 1D, and 5D harbor the highest number of detected MTAs under drought conditions (Supplementary Figure 7). Interestingly, SNP maker AX-109506123 on chromosome 5D at 528.819 Mbp exhibited a pleiotropic effect on SWD and PBW (Supplementary Table xl3). Among the 26 QTL regions, nine and four of them comprised SNP-clusters with at least two MTAs, under drought and control conditions, respectively. The other 13 QTL regions included single MTAs (Supplementary Table xl4). A hotspot of 17 MTAs in SNP-clusters associated with SDW under drought conditions was found on chromosome 7B in a chromosomic region of 32 Mbp length, while under control conditions a hotspot of 7 MTAs for KNSp was found on 5A. The genetic region on 5D from 542.108 to 546.910 Mbp was a QTL hotspot for GSC under drought comprising five MTAs in the cluster (Supplementary Figure 7).

A total of 19 QTL regions comprising 87 MTAs were significantly interacting with water regimes for seven agronomic and GQT. Among them, 10 harbor SNP-clusters, while nine QTL regions comprised each a single MTAs (Supplementary Table xl6). Plant dry biomass weight had the highest number of MTAs in SNPs-cluster on chromosome 2A (23) and 5D (16) in a region from 675.080 to 677.043 and from 559.729 to 562.834 Mbp, respectively. The SNP-cluster involved in GY was co-located with the QTL detected for PBW on chromosome 5A, which contained the highest number of interacting effect MTAs associated with GY, PBW, KN, and GPC.

### Polymorphisms in Relationship to Breeding Progress

**Figure 4:**
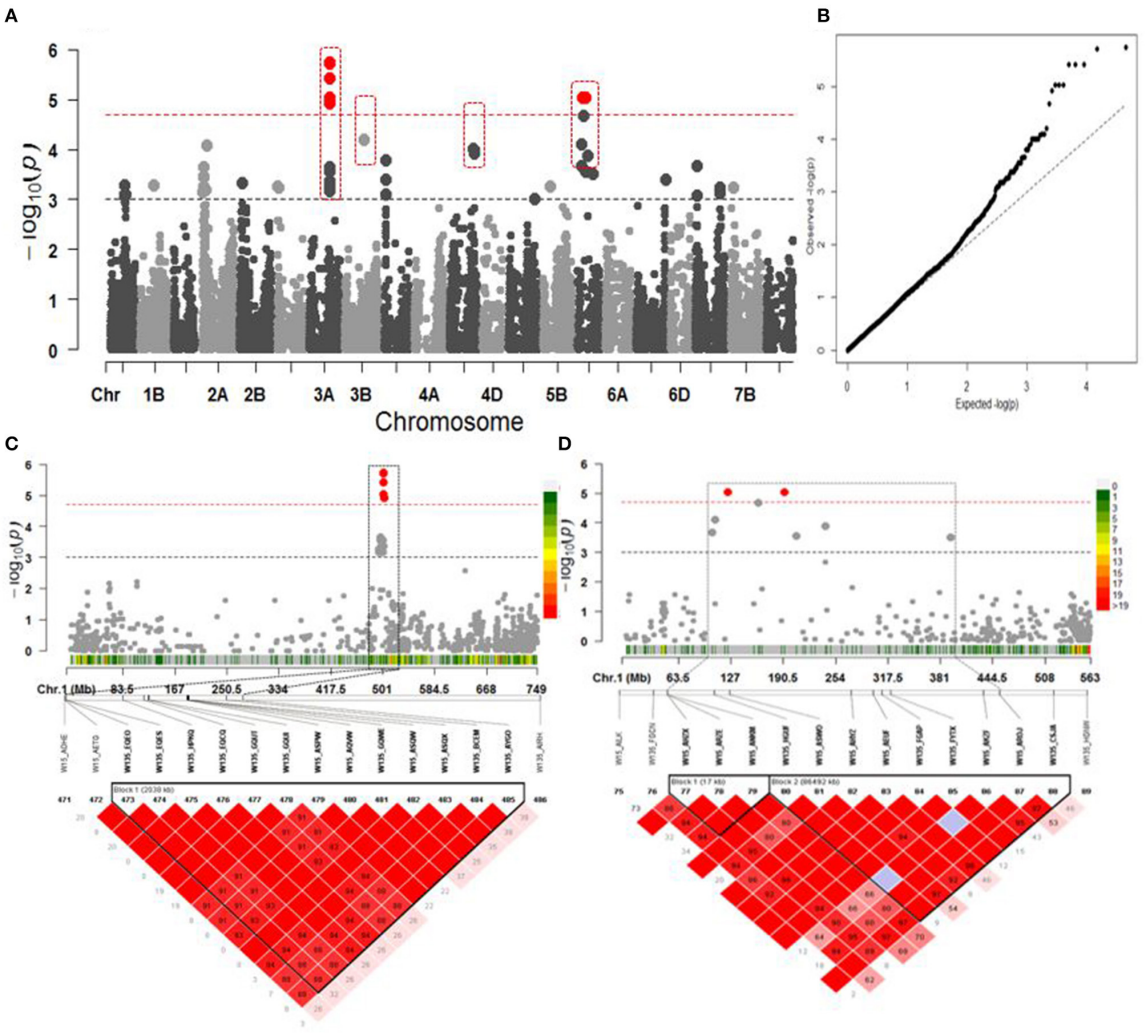
Association mapping for year of release. **(A)** Manhattan plot from association mapping using the MLM. The top 13 SNPs are shown in gray or dark gray (bigger size circle) and the SNPs exceeding the significance threshold of Q = 0.05 FDR correction are shown in red; the MTAs in dotted red squares are in common with GSC. **(B)** QQ plot of expected and observed *P*-values. **(C)** The peak region on chromosome 3A span in a region of 0.643 Mbp from 502.398 to 503.027 Mbp harbored 6 MTAs in LD block. **(D)** The peak region on chromosome 5D spanning in a region of 15.58 Mbp size had two MTAs TA002565_0478 and wsnp_Ex_rep_c67164_65655648 in the first block, while the second block of 86.492 Mbp size comprised three MTAs wsnp_Ex_c65985_64188864, wsnp_Ku_rep_c72922_72561803, and Excalibur_c10046_579. In **(C,D)**, pair-wise LD between SNP markers is indicated as *r*^2^-values: dark red indicates a value of 1 and white indicates 0. The dotted squares in **(C,D)** denote the linkage blocks that contain high significant SNPs on 3A and 5D. The color scaled legends at the right side of the Manhattans plots in **(C,D)** indicate the SNP density in a chromosomal region.

Genome-wide association studies identified 28 congruent significant MTAs comprising 12 MTAs significant at *P* <10^−4^ and 16 (*P* <10^−3^) associated with BP (Figures 4A,B). SNP markers explained from 5.86 to 11.34% of the observed phenotypic variation (*R*^2^) (Supplementary Table xl1). Among them, six and two SNPs detected on chromosomes 3A and 5D, respectively, were verified after FDR correction at Q = 0.05 (Figure 4A). The associated SNPs on 3A were in a LD block located at 500.988–503.027 Mbp (Figure 4C). The ones on 5D were located within a chromosomal region composed of two LD blocks between 107.584 and 192.270 Mbp. The first LD block covers 15.58 Mbp interval, while the second LD block is 86.492 Mbp (Figure 4D).

**Figure 5:**
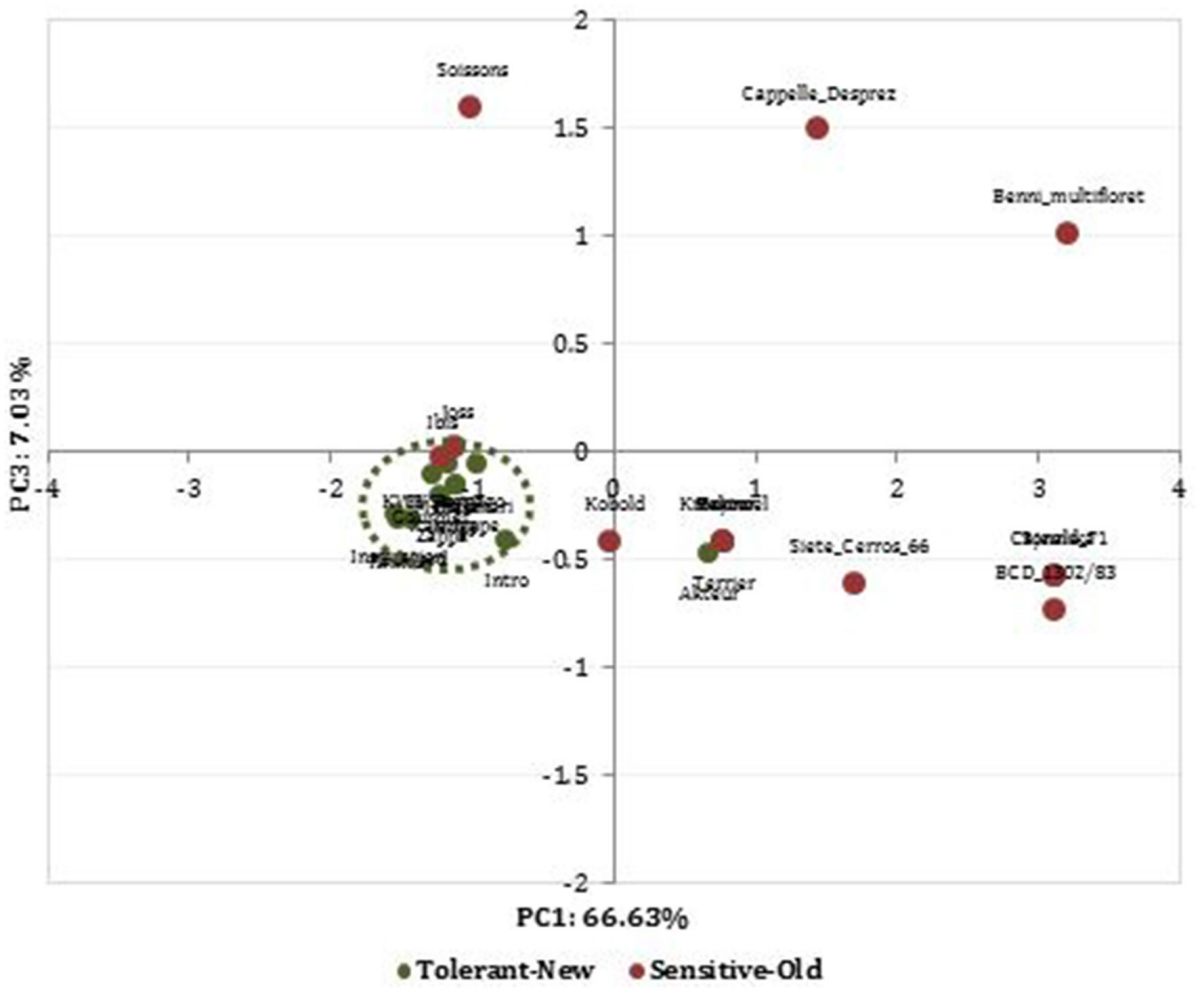
Principal component analysis (PCA) plot using a PCA matrix (Tassel 5.2) estimated with data from 30 SNPs involved in breeding progress of drought-tolerant (green color) and drought-sensitive (red color) wheat cultivars previously identified among the studied population.

We performed a PCA based these 28 identified MTAs to determine the genetic relationship among cultivars from high and low SWP values. The PCA clearly separated the wheat cultivars based on their DT status (Figure 5). Most of the recently released cultivars were drought-tolerant and belong to one group, whereas the old cultivars were the drought-sensitive. The first three PCs explained 82.75% of the observed genetic variation. The PC1 accounted for 66.63% of the variation and mostly depicted the difference between drought-tolerant new and drought-sensitive old cultivars. This component obtained higher loadings values from SNPs makers located on chromosomes 3A and 5D. The biplot PC1 vs. PC3 displayed drought-tolerant modern cultivars in the down left quadrant, whereas drought-sensitive, which were old released cultivars, were scattered randomly in the whole biplot.

### Haplotype CC Selected Through Breeding Has Enhanced Grain GSC and Drought Tolerance

**Figure 6:**
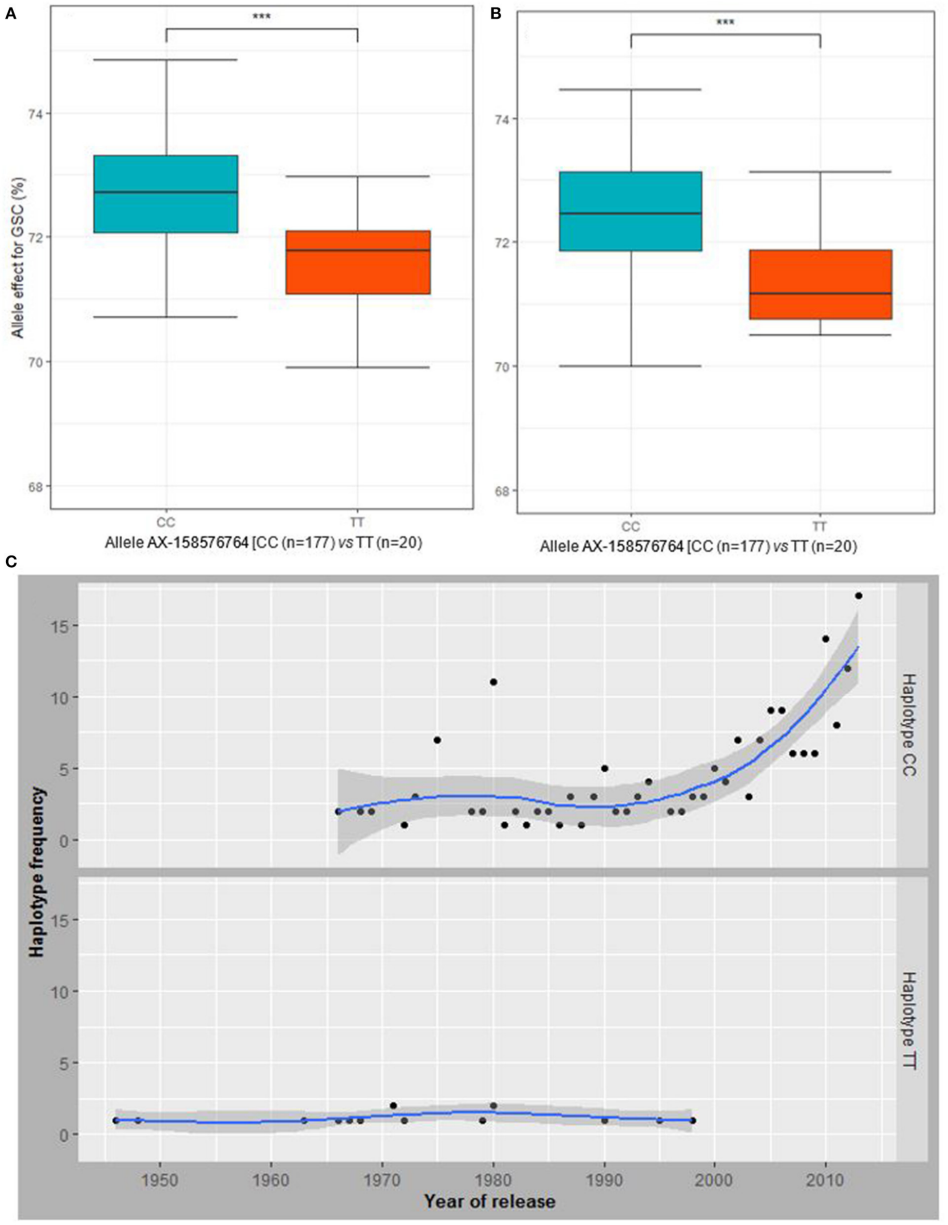
Allele AX-158576764 effect on GSC under **(A)** rainfed and **(B)** drought conditions. **(C)** The trend in the allele frequency of the haplotype block including the markers AX-158576764 and AX-111076088 over years of release of the cultivars is displaying an increase in the haplotype frequency (number of cultivars) having the favorable alleles or haplotype (CC). ***means the P-value is significant at 0.001.

Comparison of detected MTAs (*P* <10^−3^) associated with BP and the ones associated with agronomic and GQT under both water regimes revealed chromosome 3A harbors SNPs with pleiotropic effect on BP and GSC (Supplementary Table xl8). Moreover, the QTL on chromosomes 3B and 4B showed drought inducible effect and were associated with GSC under drought conditions (Supplementary Table xl8). The haplotype block on chromosome 3A located at 496.991 Mbp (Figure 4C), detected with AX-158576764 and AX-111076088 SNPs was associated with GSC under control and drought conditions (Supplementary Figure 8). The haplotype representing their major allele (CC) significantly contributed to higher GSC than the minor allele (TT) under both water regimes (Figures 6A,B). Likewise, that major allele (CC) has contributed to higher GY under drought stress. However, under rainfed conditions, the difference between both alleles of the haplotype was not significant for GY (Supplementary Figure 9). The analysis of the allele frequencies of the associated haplotype-block 3A revealed that the allele “CC” conferring higher GSC were favorably selected against the alleles “TT” that is associated with low GSC throughout the wheat breeding history (Figure 6C; Supplementary Figure 9).

### Identification of Candidate Genes Located in QTL Intervals

**Figure 7:**
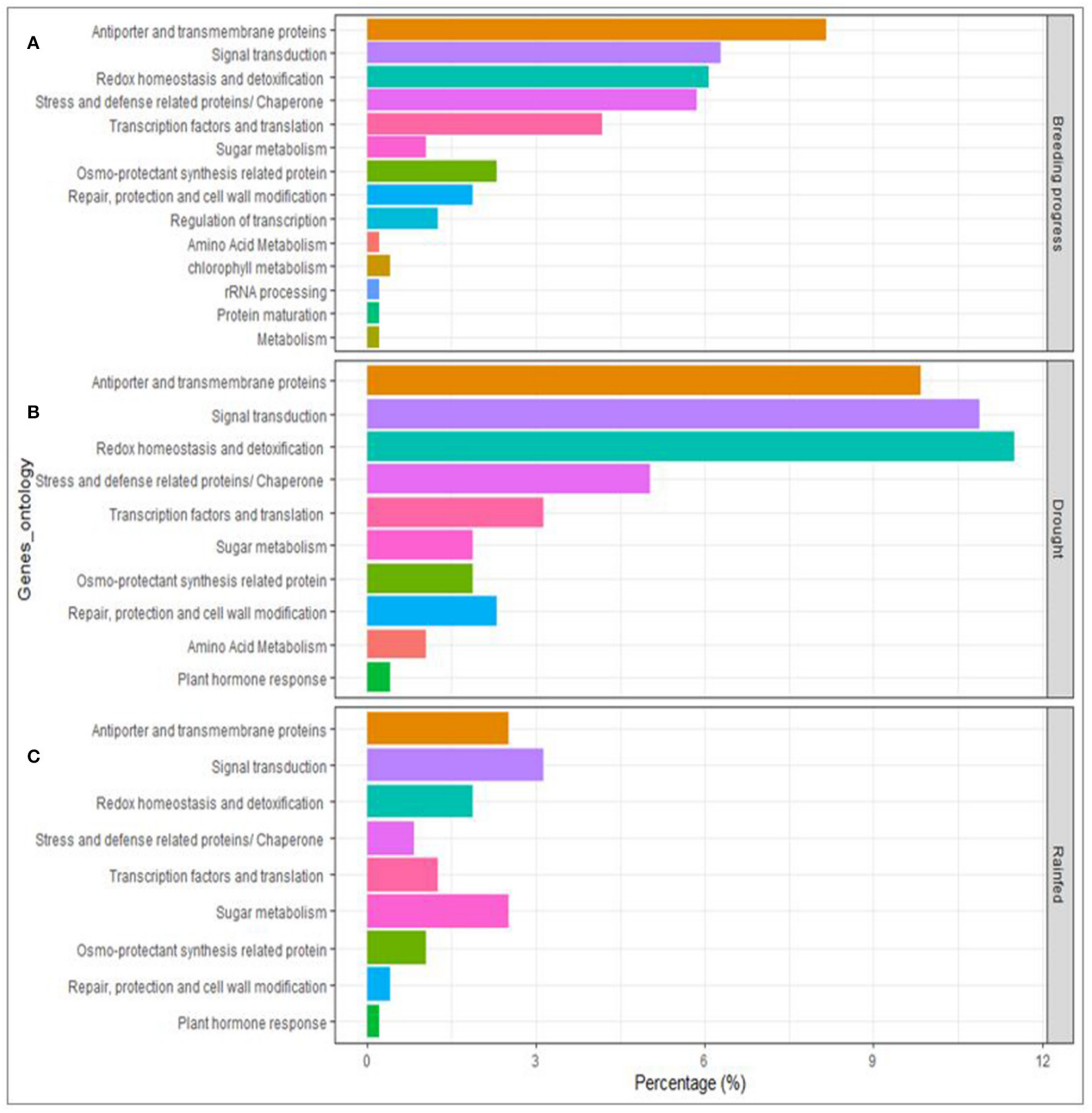
Genes annotation and ontological classifications of the associated DNA sequences underlying breeding progress and the traits of interest under drought and rainfed conditions using GWAS.

High confidence (HC) candidate genes at the vicinity of the detected SNP-clusters were retrieved from the genome assembly of *Triticum aestivum* cv. Chinese Spring. Under rainfed conditions, 94 HC genes were retrieved from six QTL regions (on 1D and 2A), whereas, under drought stress, 323 HC genes were obtained from nine QTL regions (on 4A, 4B, 4D, 5A, 5D, 7B). The chromosomal regions underlying BP contain mostly antiporters and transmembrane proteins and are enriched in genes involved signal transduction, in redox homeostasis and detoxification, and included those associated with defense mechanisms against biotic and abiotic stress. Likewise, under drought conditions, the genes category that were present for BP were also significantly detected under drought conditions. However, under rainfed conditions, those genes were not notably present in the vicinity of the detected SNPs (Figure 7).

Specifically, QTL regions underlying traits under drought stress conditions were co-located with genes involved in primary metabolism such as photosynthesis activity namely electron transport, dehydrogenase, and oxidoreductase activity (GO:0004616; GO:0016491; GO:0055114) as well as cation and zinc transporter in stress response mechanism (Supplementary Table xl5). The genetic region of chromosome 7B associated with SDW (488.412 to 520.418 Mbp) with AX-109411217 and AX-109328820 as MTAs peak harbored 177 HC genes. Under rainfed conditions, marker AX-108905462 on 5A for KNsp, AX-109506123 on 5D for PWB, and BS00101408_51 on 7B co-segregate with genes involved in molecule transport activity such as oligopeptide, heavy metal, sugar, and nucleobase ascorbate transporter, and UDP-glycosyltransferase activity.

The analyses of the genomic regions of the SNP-clusters interacting with water regimes indicated that most of the candidate genes identified in this region belong to categories of genes involved in metabolic processes (GO:0008152), transferase activity (GO:0046912), and genes encoding for drought-responsive proteins. Further, chromosomal regions on 2B and 5D associated with KN and PBW co-segregate with genes involved in disease resistance whose gene ontology (GO:0043531) terms are related to protein and ADP binding (Supplementary Table xl7).

The QTL regions on 3A, which underlaid the BP, harbors eight HC candidate genes (Supplementary Table 2), including those whose functions are related to carbohydrate metabolic process (GO:0005975), protein phosphorylation (GO:0006468), and GTPase activity (GO:0003924). The chromosomic region on 5D, which showed significant association with BP, contains 267 HC genes, including some involved in stress response mechanism (GO:0006950), disease resistance, starch synthase, and photosynthesis activity including several dehydrogenases involved in oxidoreduction process (GO:0015979) (Supplementary Table xl5).

## Discussion

The aim of this study was to evaluate the genetic variation for developmental, key yield components, and GQT, and link the observed phenotypic variation to QTL contributing to high GY, grain quality, and improved DT in wheat. To the best of our knowledge, this is the first study using different types of quantitative traits and BP information in a diverse wheat germplasm to identify drought tolerant genotypes and drought responsive QTL regions. The presented results reveal wide phenotypic variation in most of the agronomic, developmental, and GQT evaluated and the detected heritability estimates ranged from low to high. This suggests that these traits can be exploited in developing drought-tolerant wheat cultivars.

### Reduction of Cultivars Performance in Agronomic, Developmental, and Grain Quality Traits Under Drought Stress

Drought stress significantly reduced the GY by 68.71% and yield components, especially KN by 66.05% compared to control conditions. The highest impact of drought on the GY may be partly due to the cumulative effects it exerts on the yield-related traits as well as the flowering and grain filling stage (Farooq et al., 2014, Mohammadi, 2018; Sallam et al., 2019). For instance, reports indicated that drought stress caused a significant reduction in yield component traits like plant growth, SN due to early death of tillers, spike size, and TKW (Harris et al., 2002; Ozturk and Aydin, 2004; Daryanto et al., 2016). Following heading, prolonged drought can reduce the pollination of the ovary because of an increased ABA concentration in the spike, leading to an increased seed abortion and thus to a reduced seed set (Weldearegay et al., 2012). It is also known that drought can cause significant limitations during grain filling due to reduced net photosynthesis caused by oxidative damage to chloroplasts and stomatal closure (Farooq et al., 2014). As an example of limitations, we observed that drought stress has reduced time to reach growth stages, hence it has stimulated plant growth, which negatively impacted GY as reported in several previous studies (Barnabás et al., 2008; Munjonji et al., 2016; Sukumaran et al., 2018). Although, we did not measure the grain filling duration, the drought stress imposed at early growth stage may have reduced this stage, thus GY more in 2017 than in 2018 under drought conditions. The reduction in GY and yield-related traits under drought stress is a common phenomenon and is controlled by several complex molecular, physiological, and morphological factors across plant growth stages (Kadam et al., 2018; Mohammadi, 2018).

In the present study, drought had negative effect on GSC, as already reported (Barnabás et al., 2008), and also on GPC. Generally, drought stress reduces starch accumulation and increases the protein content (Flagella et al., 2010). The decrease of GPC detected in our study may be due to the application of drought at very early stage of plant development. Indeed, it has been reported that the effect of drought stress on grain quality highly depends on its intensity and when it occurs (Rakszegi et al., 2019). Larger phenotypic variations were observed among the wheat cultivars under drought stress when compared to the rainfed conditions as indicated by higher CV and more dispersed scatter points across regression lines. That would suggest the existence of substantial genotypic differences in the response to drought in the studied population. This high genetic diversity is a valuable resource providing the fundaments for future breeding for DT (Frei, 2015; Oyiga et al., 2016). Under rainfed field, it was not obvious to detect visually the difference between the genotypes for their developmental traits. Contrary to that, under drought conditions, a clear estimation of the genotypes' response to drought was possible. The visual scored developmental traits showed the highest CV in the study, hence confirmed the existence of huge genetic variation when plants are under stress conditions as reported (Oyiga et al., 2016). The lower heritability values observed under drought compared to rainfed conditions reflect the higher variation among repetitions. Also, the heritability calculated across treatments was generally lower compared to heritability within treatments. That could be explained by the significance difference between genotypes performance under drought and rainfed conditions.

The correlation between GY and KN was higher under drought than under rainfed. Monneveux et al. (2012) reported that KN is the most relevant trait among yield components contributing to high GY. The highest slope from the regressions GY vs. KN was found under drought conditions, suggesting the increase of KN would enhance more the GY under drought than under rainfed conditions. Moreover, an increase in the grain starch correspondingly increased the GY, particularly under drought stress. Thus, could serve as an important proxy when breeding for DT. High starch deposition could be connected to higher photosynthetic activity and photosynthates assimilation, which would increase KN and consequently GY. Starch availability is essential during embryo development, and sufficient starch greatly increases the number of fertile floret, hence the KN (Boyer and Westgate, 2004). Our finding of lower correlation between TKW with GY observed under drought conditions compared to rainfed has been previously reported by Del Pozo et al. (2016) and that could be due to the decrease of TKW under drought conditions. Neutral detergent fiber showed negative correlation with GPC, and positive association with GSC under both water regimes, but inconstantly associated with agronomic traits across water regimes. Drought effect on NDF was not significant in 2017, but it significantly decreased this nutritional parameter in 2018 in which no genotypic effect was observed. The effect of drought on fiber utilization by animals are less clear and limited (Vincent et al., 2005; Ferreira and Brown, 2016; Ferreira et al., 2021).

The present study showed that the relative values of leaf greenness were positively associated with the LRr which is due to the loss of cell turgor pressure in leaves. Both traits were highly correlated with GY under drought treatment. The stay green of flag leaf provides insights on the ability of leaves to remain photosynthetically active due to delayed senescence (Thomas and Howarth, 2000), and has been reported to be highly correlated with WUE during grain development and with GY under drought conditions (Christopher et al., 2016). Cultivars with prolonged stay green ability are high yielding because up to 50% of the photosynthates needed during grain filling are contributed by flag-leaf photosynthesis (Sylvester-Bradley et al., 1990; Larbi and Mekliche, 2004).

### Breeding Contribution to Cultivars Performance and Drought Tolerance

Contrary to the belief that crop improvement has reduced their potential to adapt to future challenges such as drought (Byrne et al., 2018; Swarup et al., 2020), our results showed that breeding has improved cultivars performance under both water regimes. Considering that the germination of all cultivars was above 95%, we concluded that the difference between older and newer released cultivars for SN per meter square was due to their performance under drought conditions. We discovered that breeding has increased the KN, HI, and GY production under both rainfed and drought conditions as previously reported (Royo et al., 2007). Drought-tolerant cultivars differed from sensitive ones by showing higher performance under drought conditions, hence having higher SWP values. Interestingly, most of the identified drought-tolerant cultivars are the recently released cultivars. They showed high yielding potential than older cultivars under drought stress conditions. Reports have also shown that modern cultivars are higher yielding compared to older ones under low nitrogen application owing to accumulated genetic variants conferring favorable effects on key yield traits (Slafer and Araus, 2007; Voss-Fels et al., 2019). Breeding has improved yield potential under optimum conditions as well as under stressful conditions through developing semi-dwarf cultivars with reduced PH, which has improved resource allocation and increased green canopy duration (Lichthardt et al., 2020). Under rainfed conditions, the BP for GY was low, whereas Voss-Fels et al. (2019) found high BP for this trait under both limited conditions (drought, low agrochemical inputs) and optimal conditions (irrigated, high agrochemical inputs) using the same wheat panel. The low BP obtained for GY under rainfed in the current experiment may be due to the small plot size, which in the absence of any stress may not favor detection of differences, as shown by low CV under rainfed than under drought conditions. The BP on GPC was decreasing over years as reported in Voss-Fels et al. (2019). using the same panel. However, they found an increase of the total protein content per ha over year of release.

### Marker Traits Association and SNP Clustering

The association mapping identified 25 and 53 MTAs under rainfed (PVE = 6.84–10.27%) and under drought (PVE = 6.12–11.29%) conditions, respectively. The higher PVE recorded under drought is indicating that the related genes are explaining more the observed variation under this condition than under control. This suggests that breeding for drought prone environment using genetic markers is achievable and promising to improve GY (Kumar et al., 2008; Mohammadi et al., 2014). The threshold for significant SNP set *P* <10^−4^ enabled the identification of SNPs with strong effects on evaluated traits. SNP-clusters under drought carried more MTAs than rainfed conditions, indicating an activation of great variety of genes with synergistic effect (Yang et al., 2010). As previously reported, drought stress is a major external stimuli that causes the overproduction of oxidative reactive oxygen species (ROS), which leads to the disruption of cells membrane integrity and later reduction in plant growth (Mohammadi, 2018). Plants respond to drought stress by producing several antioxidant enzymes such as catalase (CAT), ascorbate peroxidase (APX), guaiacol peroxidase (GPX) playing important role in ROS scavenging (Dudziak et al., 2019).

We found on 4B (marker AX-110400483), a QTL affecting PH. The homeologous locus on 4D, that led to a reduction of PH has been recently reported (Alqudah et al., 2020). Likewise, the haplotype block on chromosome 4B including SNP markers associated with BP, has reducing effect on PH and TKW, but increased GSC and yield. The chromosome 4B and 4D have been reported to harbor the genes Rht-B1b (formerly Rht1) and Rht-D1b (Rht2) in wheat (Börner et al., 1996; Hedden, 2003).

### Genetic Regions With Hotspot QTL Affecting Multiple Traits and Related Candidate Genes

The GWAS performed revealed that the QTL region on chromosome 3A has a pleiotropic effect on BP and GSC. QTL regions for GQT such as seed loaf volume and crumb quality were identified on chromosome 3A (Kuchel et al., 2006). It has been reported that that chromosome 3A played an important role in wheat yield and harbors genes related to morphological and physiological traits such as tiller inhibition, a shoot architecture influencing trait (Araus et al., 2008; Kuraparthy et al., 2008; Czyczyło-Mysza et al., 2011; Farooq et al., 2014). The *in silico* analyses showed that this region located at 500.988–503.027 Mbp interval contains eight HC genes, whose biological functions specify them as the probable candidate genes for the observed drought stress response (Supplementary Table xl5). These genes were found to regulate carbohydrate metabolic process, protein phosphorylation, and GTPase activity, etc. in wheat/or plant species, and might play a role in higher starch content in newer released genotypes. Likewise, some transcription factors like WRKY which mediates several abiotic stress responses (Phukan et al., 2016) and RING binding protein genes affecting ubiquitin protein ligase activity (GO:0005515; GO:0008270) were identified in the same chromosomic region.

QTL region on chromosome 5A spanning from 586.153 to 589.296 Mbp with the peak marker AX-108905462, which included a hotspot MTAs for KNSp under rainfed conditions, has been previously reported to have an association with leaves bronzing score (LBS) and ozone tolerance (Begum et al., 2020). QTL mapped for LBS of rice under ozone stress positively affected agronomic traits such as GY (Wang et al., 2014) and grain quality (Jing et al., 2016). Previous studies revealed the association of chromosomic 5A region to KNSp, GY, and flag-leaf rolling index (Czyczyło-Mysza et al., 2011; Farooq et al., 2014). Therefore, the highest MTAs hotspot under rainfed in our study could be of high interest to increase the number of the kernel per spike, which has an important effect on wheat yield.

The linkage block on 5D detected also for BP has been reported as a region harboring QTL associated with KNSp, TKW, and GY (Czyczyło-Mysza et al., 2011; Farooq et al., 2014). This linkage group co-segregates with genes involved in photosynthesis activity such as protein disulfide oxidoreductase activity, electron carrier activity, and contains PSII reaction center protein complex that produces the ATP and reduces the NADP+ to NADPH. Both ATP and NADPH are converted into glucose in the light-independent reaction of photosynthesis (Shi and Schröder, 2004). Reduction of net photosynthesis caused by oxidative damage to chloroplasts and stomatal closure under drought (Farooq et al., 2014) can cause significant limitations during grain filling, hence a limiting factor of higher yield. However, the activation of various drought responsive genes under enable some wheat genotypes to maintain physiological activities (Yue et al., 2006; Luo, 2010) and tolerate drought stress. The identified drought-responsive QTL regions and related candidate genes unraveled in our study should warrant further investigation as they may facilitate the molecular breeding of drought-tolerant wheat, thereby contributing to global food security.

## Conclusion

The present study identified KN as the key component that importantly contributes to GY under drought stress conditions and uncovered genetic loci underlying GY under drought stress and rainfed conditions. The high density of SNPs mapped across the 21 chromosomes has enabled the identification with precision (<10 Mbp) the genetic region associated with traits of interest. SNP-clustering approach was useful to identify chromosomal regions harboring QTL hotspots of MTAs with synergic effects. Our findings demonstrated the existence of huge genetic variation in the evaluated germplasm that could be used to develop drought-tolerant cultivars. Cultivar performance particularly for GY has been increased by breeding under rainfed and drought conditions through improving key yield components such as SN and KN, and incrementing favorable alleles for high grain starch accumulation, which afterward positively affects wheat yield. Breeding has contributed to conserve genomic regions that contain important genes playing role in detoxification against oxidative stress and in defense mechanisms against drought stress. Upon validation, these favorable alleles regulating these traits can be effectively used in breeding programs to improve yield under drought-prone environments.

## Data Availability Statement

The original contributions presented in the study are included in the article/Supplementary Materials, further inquiries can be directed to the corresponding author/s.

## Author Contributions

AK performed experiments, data analyses, and drafted the manuscript. AK and MB performed the data collection. AB, AK, and JL designed the experiments and interpreted the results. AB, BO, and JL were responsible for the correction and critical revision of the manuscript. All authors read and approved the final manuscript.

## Conflict of Interest

The authors declare that the research was conducted in the absence of any commercial or financial relationships that could be construed as a potential conflict of interest.

## Publisher's Note

All claims expressed in this article are solely those of the authors and do not necessarily represent those of their affiliated organizations, or those of the publisher, the editors and the reviewers. Any product that may be evaluated in this article, or claim that may be made by its manufacturer, is not guaranteed or endorsed by the publisher.
