# Supplementary material for: Breeding Driven Enrichment of Genetic Variation for Key Yield Components and Grain Starch Content Under Drought Stress in Winter Wheat
Source: Front Plant Sci. 2021 Aug 16;12:684205. doi: 10.3389/fpls.2021.684205 (PMC8415485; doi:10.3389/fpls.2021.684205)
Supplement: Supplementary file 1 [file Data_Sheet_1.docx]

# Supplementary Material

## **TABLE S1 A|** Complete description and abbreviation of evaluated traits in the study

| **Traits** | **Abbreviation** | **Descriptions and units** |
| --- | --- | --- |
| ***Agronomic traits*** | | |
| Plant height | PH | Plant height was measured from three different plants of every plot at physiological maturity from the soil surface to the tip of the head, excluding awns (cm) and mean values were generated for further analysis. |
| Grain yield | GY | The plots were harvested and the grains cleaned, weighed and estimated in g/row. |
| Shoot dry weight | SDW | Dry shoot weight (g/row), dried shoot sample were put in oven set at 65°C for 3 days and the weight was measured. |
| Aboveground plant biomass weight | PBW | After drying, plant biomass including GY (PBW) was recorded to estimate the HI as the ratio of GY to total plant biomass weight. |
| Spike number | SN | Spike numbers were counted for a genotype within 90 centimeter row for all genotypes one by one before harvesting, and were used to calculated the SN per meter square. |
| Kernels number | KN | Grain number per meter square, grain number per spike were calculated based on the thousand kernel weight, spike numbers per meter square, grain weight per meter square. |
| Kernels number per spike | KNSp |  |
| Harvest index | HI | HI =GY/ PBW similar to HI= GY/ (SDW+GY) |
| Thousand kernel weights | TKW | Weight of a thousand well developed whole grains dried sample (g). Three replicates were used to calculate the mean. |
| ***Developmental traits*** | | |
| Relative plant healthiness | HSr | The score of each trait was given with the customized scale from 1 to 5 equivalent to 0, 25, 50, 75 and 100% of damage, where the score 1 was 0 % of bad phenotype indicating the best performance while 100% of bad phenotype was the full expression of worst performance (Pask et al., 2012).  For example, the leaf greenness was scored as follows: 1 means 0 % leaves were yellow and 100% were green; 2 means 25% leaves were yellow, 75% leaves were green, and 5 means 100% leaf are yellow. For leaf rolling and greenness, observations were done on the flag leaves and second youngest leaves. The relative value of each developmental trait was calculated as the inverse function of the visual scored value. The greater the relative value, the higher was the performance of the genotype. |
| Relative plants homogeneity of growth | HGr |  |
| Relative plant leaves greenness | LGr |  |
| Relative plant leaves rolling state | LRr |  |
| ***Grain quality*** | | |
| Grain protein content ratio | GPC | The grain quality were analyzed using NIRS instrument (Perten, DA 7250) following the manufacturers guidelines (the values are given in %) |
| Grain starch content ratio | GSC |  |
| Neutral detergent fiber ratio | NDF |  |

## **TABLE S1 B |** Duration of developmental growth stages

| Year | Sowing date | Starting of drought (in DAS) | Havesting date (*****DAS) | | Duration prebooting (Start of drought) to harvesting | | Growth stage | Duration (days after sowing, DAS Anova**^b^**) | |
| --- | --- | --- | --- | --- | --- | --- | --- | --- | --- |
|  |  |  | Ds | Rf | Ds | Rf |  | Ds | Rf |
| 2017 | 29.11.2016 | 15.04.2017 (137) | 28.06.2017 (211) | 28.07.2017 (241) | 74 | 104 | Prebooting |  | 170 |
|  |  |  |  |  |  |  | Booting | 170 | 175 |
|  |  |  |  |  |  |  | Heading | 174 | 187 |
|  |  |  |  |  |  |  | Anthesis | 178 | 191 |
|  |  |  |  |  |  |  | Fruit Development | 191 |  |
| 2018 | 02.11.2017 | 14.04.2018 (163) | 03.07.2018 (243) | 19.07.2018 (259) | 80 | 96 | Prebooting | 188 | 191 |
|  |  |  |  |  |  |  | Booting | 189 | 197 |
|  |  |  |  |  |  |  | Heading | 192 | 197 |
|  |  |  |  |  |  |  | Anthesis | 191 | 217 |
|  |  |  |  |  |  |  | Fruit Development | 218 |  |

DAS means date after sowing

***** Duration from sowing to harvesting;

**^b^** Anova of water regime on developmental traits duration

Water Regime treatment T (P<0.001)

Growth stage GS (P<0.001)

Interaction W*GS (P<0.001)

## **TABLE S2 |** ANOVA and descriptive statistics developmental (Dev) traits of 200 wheat genotypes evaluated in two water regimes across 2017 and 2018 growing seasons

| Developmental traits | | | | | | | | | |
| --- | --- | --- | --- | --- | --- | --- | --- | --- | --- |
|  | Drought stress 2017 | | | |  | Drought stress 2018 | | | |
| Statistics | HSr | HGr | LGr | LRr |  | HSr | HGr | LGr | LRr |
| Mean | 0.811 | 0.634 | 0.475 | 0.459 |  | - | - | 0.617708 | 0.714052 |
| CV (%) | 30.07 | 37.08 | 45.26 | 55.86 |  | - | - | 42.38 | 35.91 |
| Heritability | - | 0.29 | 0.25 | 0.21 |  | - | - | 0.43 | 0.28 |
| G effect | ns | ** | ** | * |  | - | - | *** | ** |

The abbreviations of traits names are given in Table S1. CV means coefficient of variation. The significance level *P<0.05, **P<0.01, ***P<0.001.

## **TABLE S3 |** Multiple linear regression of GY *vs* evaluated traits under rain fed and drought stress conditions during 2017 and 2018. Note that PBW and HI were not included in the regression because PBW is the sum of SDW and GY, and not an independent component. HI is the ratio of GY to PBW weight.

| Years | Treatment | Control | | | Drought | | |
| --- | --- | --- | --- | --- | --- | --- | --- |
| Statistics | | Pvalue | Sig | Adj. R-squared (%) | Pvalue | Sig | Adj. R-squared(%) |
| 2017 | PH | 0.0449 | * | 78.85 | 0.269 |  | 78.92 |
|  | SN | <0.001 | *** |  | <0.001 | *** |  |
|  | KN | <0.001 | *** |  | 0.036978 | * |  |
|  | KNSp | <0.001 | *** |  | <0.001 | *** |  |
|  | SDW | <0.001 | *** |  | <0.001 | *** |  |
|  | TKW | <0.001 | *** |  | <0.001 | *** |  |
|  | NDF | 0.885 |  |  | 0.638 |  |  |
|  | GPC | 0.033 | * |  | 0.791 |  |  |
|  | GSC | 0.451 |  |  | 0.073 |  |  |
|  | HSr | - |  |  | 0.831 |  |  |
|  | HGr | - |  |  | 0.055 |  |  |
|  | LGr | - |  |  | 0.267 |  |  |
|  | LRr | - |  |  | 0.059 |  |  |
| 2018 | PH | 0.048 | * | 96.17 | 1.000 |  | 98.08 |
|  | SN | <0.001 | *** |  | 0.436 |  |  |
|  | KN | <0.001 | *** |  | <0.001 | *** |  |
|  | KNSp | <0.001 | *** |  | 0.983 |  |  |
|  | SDW | 0.165 |  |  | 0.692 |  |  |
|  | TKW | <0.001 | *** |  | <0.001 | *** |  |
|  | NDF | 0.892 |  |  | 0.838 |  |  |
|  | GPC | 0.221 |  |  | 0.110 |  |  |
|  | GSC | 0.838 |  |  | 0.292 |  |  |
|  | HSr | - |  |  | - |  |  |
|  | HGr | - |  |  | - |  |  |
|  | LGr | - |  |  | 0.678 |  |  |
|  | LRr | - |  |  | 0.228 |  |  |

The significance level *P<0.05, **P<0.01, ***P<0.001; The abbreviations of traits names are given in Table S1.

## **TABLE S4 |** Summary statistics of breeding progress (absolute and relative) of GY related traits and grain quality for 2017 and 2018 growing seasons together (Lsmeans) and the type of dynamic in the ABP.

| Tratits | Lsmeans | | | | | | | | | | | | | |
| --- | --- | --- | --- | --- | --- | --- | --- | --- | --- | --- | --- | --- | --- | --- |
|  | Control | | | | | |  | Drought | | | | | | Pattern Types**^α^** |
|  | Mean | Mean | Sig (p) | ABP (slope) | R² | RBP (%) |  | Mean | Mean | Sig (p) | ABP (slope) | R² | RBP |  |
|  | Oldest | Newest |  |  |  |  |  | Oldest | Newest |  |  |  |  |  |
| PH (cm) | 87.11 | 78.23 | *** | -0.24 | 0.22 | -7.59 |  | 69.82 | 65.01 | *** | -0.14 | 0.15 | -5.77 | Types II |
| GY (g/row) | 226.67 | 238.54 | ns | 0.067 | 1E^-3^ | 0.83 |  | 65.07 | 76.42 | *** | 0.3 | 0.09 | 12.16 | Types I |
| SDW (g/row) | 251.06 | 230.76 | *** | -0.87 | 0.14 | -9.41 |  | 96.47 | 96.72 | ns | -0.052 | 2.3E^-3^ | -1.46 | Types II |
| PBW (g/row) | 476.62 | 469.30 | ** | -0.8 | 0.05 | -4.65 |  | 162.19 | 173.26 | ns | 0.25 | 0.02 | 4.55 | Types III |
| TKW (g) | 43.97 | 42.81 | ns | -0.03 | 0.02 | -1.91 |  | 39.22 | 38.19 | * | -0.03 | 0.02 | -2.41 | Types II |
| SN (per m^2^) | 750.28 | 737.01 | * | -0.75 | 0.02 | -3.15 |  | 338.07 | 365.91 | * | 0.57 | 0.05 | 5.2 | Types III |
| KN (per m^2^) | 28152.74 | 30184.73 | ns | 26 | 0.01 | 2.74 |  | 9394.34 | 10895.8 | ** | 32 | 0.04 | 9.27 | Types I |
| KNSp | 38.65 | 41.95 | ** | 0.07 | 0.04 | 4.91 |  | 26 | 28.84 | ** | 0.06 | 0.04 | 7.07 | Types I |
| HI | 0.48 | 0.51 | *** | 7.6E^-4^ | 0.16 | 4.52 |  | 0.39 | 0.43 | *** | 9.3E^-4^ | 0.11 | 6.32 | Types I |
| GPC | 14.58 | 14.05 | ** | -0.01 | 0.04 | -1.86 |  | 13.67 | 12.89 | *** | -0.02 | 0.11 | -4.37 | Types II |
| GSC | 72.31 | 72.92 | ** | 0.01 | 0.04 | 0.46 |  | 71.85 | 72.72 | *** | 0.02 | 0.08 | 0.78 | Types I |
| NDF | 18.09 | 18.38 | ** | 0.01 | 0.05 | 2.07 |  | 17.97 | 18.31 | ** | 0.01 | 0.05 | 2.05 | Types I |

**^α^** The regression results (Figure 2, Figure S5) revealed three types of patterns in the absolute breeding progresses when comparing the slopes of control and drought treatments as indicated in Table S4. The pattern type I is when breeding has increased genotypes achievements under both water regimes. In this group were found GY (Figure 2A) and its key components, namely KN (Figure 2B) and KNSp (Figure S5E), HI (Figure 2C), GSC and NDF (Figure S5HI); Type II means negative slopes, which indicated a decrease in trait performance brought by breeding (SDW, PH, TKW and GPC in Figure S5ACFG), while Type III indicated a positive slope under drought and a negative slope under control condition. * P-value tests the significance of the slope. While the third pattern included PBW SDW, and SN (Figure S5ABD), where breeding has increased genotypes performance under drought but reduced it under control.

Means oldest and newest are the average value of genotypes released before 1980 and after 2010, respectively. Absolute (ABP) and relative (RBP) breeding progress were derived from regression models. Sig (p) gives the significance level of the slopes (*** means significant at 0.001, *** at 0.01 and * at 0.05 and ns. indicates not significant at 0.05). Relative breeding progress is expressed in percent (%).

## **TABLE S5 |** Pairwise comparison of regressions coefficients (intercepts and slopes) of model GY *vs* yield components, and traits of interest *vs* year of release under both water regimes.

| Traits | *Intercepts^+^* | *Slopes^+^* |
| --- | --- | --- |
| *GY vs Yield components* | | |
| SDW | 7.9e-14 *** | 0.834 ns |
| KNSp | 2.96e-14 *** | 0.324 ns |
| KN | 0.000381 *** | 0.038531 * |
| SN | 2.38e-08 *** | 0.433 ns |
| TKW | 0.107211 | 0.000206 *** |
| *Traits vs Year of release* | | |
| PBW | 6.82e-05 *** | 0.000497 *** |
| SDW | 6.19e-07 *** | 4.50e-06 *** |
| GY | 0.0506 | 0.1485 ns |
| KN | 0.451 ns | 0.771 ns |
| KNSp | 0.8916 ns | 0.9508 ns |
| HI | 0.3748 ns | 0.4826 ns |
| PH | 0.0103 * | 0.0173 * |
| SN | 0.000354 *** | 0.001800 ** |
| TKW | 0.885043 ns | 0.973138 ns |
| GPC | 0.08446 | 0.06561 |
| GSC | 0.2141 ns | 0.2196 ns |
| NDF | 0.98461 ns | 0.98461 ns |

*^+^* the numbers are displaying the P values from the comparisons.

## **TABLE S6 |** Drought-tolerant and Drought-sensitive genotypes identified based on the SWP values of agronomic, developmental and grain quality (GQ) traits.

| Tolerant | | | | | |  | Sensitive | | | | | |
| --- | --- | --- | --- | --- | --- | --- | --- | --- | --- | --- | --- | --- |
| Entry name | Release Year | Agro | Dev | GQ | Times selected |  | Entry name | Release Year | Agro | Dev | GQ | Times selected |
|  |  |  | Traits |  |  |  |  |  |  | Traits |  |  |
| Claire | 1999 | 1 |  |  | 1 |  | Estivus | 2012 | 1 | 1 |  | 2 |
| Zappa | 2009 | 1 |  | 1 | 2 |  | Kronjuwel | 1980 | 1 |  |  | 1 |
| Meister | 2010 | 1 |  |  | 1 |  | Mulan | 2006 | 1 | 1 |  | 2 |
| KWS Santiago | 2011 | 1 | 1 |  | 2 |  | Solstice | 2001 | 1 |  |  | 1 |
| Brigand | 1979 | 1 |  | 1 | 2 |  | Arktis | 2010 |  | 1 | 1 | 2 |
| Edward | 2013 | 1 |  |  | 1 |  | Joss | 1972 | 1 |  |  | 1 |
| Jenga | 2007 | 1 |  | 1 | 2 |  | Rektor | 1980 | 1 |  |  | 1 |
| TJB 990-15 | 1980 | 1 | 1 | 1 | 3 |  | Cappelle Desprez | 1946 | 1 |  |  | 1 |
| Gourmet | 2013 | 1 | 1 |  | 2 |  | Tiger | 2001 | 1 |  |  | 1 |
| Kalahari | 2010 | 1 |  |  | 1 |  | Ibis | 1991 | 1 |  |  | 1 |
| Intro | 2011 | 1 | 1 |  | 2 |  | Aszita | 2005 | 1 |  | 1 | 2 |
| Primus | 2009 |  | 1 | 1 | 2 |  | Kobold | 1978 | 1 |  | 1 | 2 |
| Inspiration | 2007 | 1 |  |  | 1 |  | Benno | 1973 | 1 | 1 |  | 2 |
| SY Ferry | 2012 | 1 | 1 |  | 2 |  | Benni multifloret | 1980 | 1 |  | 1 | 2 |
| Terrier | 2001 | 1 |  |  | 1 |  | Caphorn | 2000 | 1 |  | 1 | 2 |
| Xanthippe | 2011 | 1 |  | 1 | 2 |  | Soissons | 1987 |  | 1 | 1 | 2 |
| Knirps | 1985 | 1 |  |  | 1 |  | BCD 1302/83 | NA | 1 |  | 1 | 2 |
| Akteur | 2003 | 1 | 1 |  | 2 |  | Sonalika | 1967 | 1 | 1 | 1 | 3 |
| Diplomat | 1966 | 1 |  |  | 1 |  | Cajeme 71 | 1971 | 1 | 1 |  | 2 |
| Basalt | 1980 | 1 | 1 |  | 2 |  | Siete Cerros 66 | 1966 | 1 | 1 | 1 | 3 |
| Average Release year | 2000.8 |  | | | |  | Average Release year | 1985.6 |  | | | |
|  |  |  |  |  |  |  |  |  |  |  |  |  |
|  |  |  |  |  |  |  |  |  |  |  |  |  |
|  |  |  |  |  |  |  |  |  |  |  |  |  |

## **TABLE S7 |** Summary of SNP markers significantly associated with evaluated traits under both water regimes.

| **Traits** | **Rainfed** | | **Drought** | | **Both Water regimes** | |
| --- | --- | --- | --- | --- | --- | --- |
|  | **MTAs number** | **MTAs R^2^ (%)** | **MTAs number** | **MTAs R^2^ (%)** | **MTAs number** | **MTAs R^2^ (%)** |
| **PH** | 5 | 8.13 | 14 | 7.68 | 19 | 7.80 |
| **GY** |  |  | 2 | 8.45 | 2 | 8.45 |
| **PBW** | 1 | 7.44 | 1 | 9.63 | 2 | 8.54 |
| **SDW** | 1 | 8.11 | 23 | 8.76 | 24 | 8.74 |
| **HI** |  |  | 1 | 7.84 | 1 | 7.84 |
| **KNSp** | 17 | 8.41 | 5 | 8.80 | 22 | 8.50 |
| **SN** |  |  | 2 | 7.29 | 2 | 7.29 |
| **TKW** | 1 | 7.65 |  |  | 1 | 7.65 |
| **GSC** |  |  | 5 | 7.13 | 5 | 7.13 |
| **Grand Total** | **25** | **8.27** | **53** | **8.26** | **78** | **8.26** |

## **TABLE Sxl1 |** Full description of MTAs for evaluated traits including 78 MTAs and 253 significant at P<10^-4^ and P<10^-3^, respectively, and 12 MTAs (P<10^-4^) and 17 (P<10^-4^) for year of release

## **TABLE Sxl2 |** Full description of MTAs (P<10^-4^) underlying evaluated traits across both water regimes

## **TABLE Sxl3 |** Chromosomal LD and information on the SNP density across genomes

## **TABLE Sxl4 |** SNP cluster or QTLs regions associated with evaluated traits

## **TABLE Sxl5 |** Candidate genes in the regions of MTAs–clusters associated to a trait

## **TABLE Sxl6 |** Information on MTAs–clusters interacting with water regimes, and the majors and minor alleles average values.

## **TABLE Sxl7 |** Candidate genes in the regions of MTAs–clusters interacting with water regimes

## **TABLE Sxl8 |** Pleiotropic SNP for GSC under both water regimes across two growing seasons.

## **TABLE Sxl9 |** Phenotypic data BLUEs values under two water regimes.


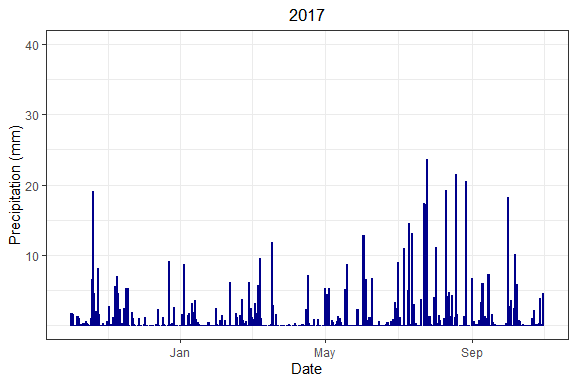

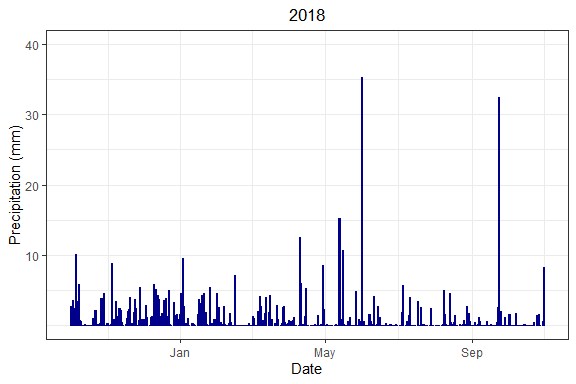

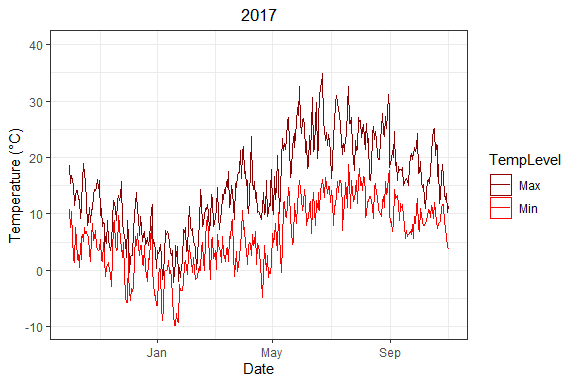

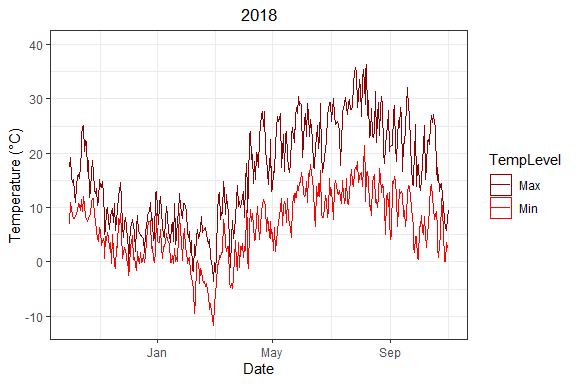


**B**

**C**

**A**

**D**

## **FIGURE S1 |** Above ground and underground weather data. **(A)** Graph of minimum and maximum temperature and daily precipitation sum during the experimental periods of 2017 and 2018; **(BCD)** Soil moisture content and soil temperature (0–30 cm depth) at latter stress time point under rainfed and drought stress of the experimental plots in the 2017 and 2018 growing seasons

**B**

**A**

**C**

**D**

## **FIGURE S2 |** Pearson correlation coefficients between evaluated traits in 2017 under rain fed field **(Panel A)** and drought stress **(Panel B)** and in 2018 under rain fed field **(Panel C)** and drought stress **(Panel D)** conditions. Phenotypic traits with their histograms are given in the diagonal panel. Lower diagonal panel represents the scatter plot with red line depicting the best fit. The upper panel represents the Pearson correlation coefficient value and size of the correlation coefficient is proportional to the strength of the correlation. The correlation coefficient significance level *P<0.05, **P<0.01, ***P<0.001. The abbreviations of traits names are given in Table S1

## **FIGURE S3 |** The principal component analysis of evaluated traits with first t wo principal components (PC1 and PC2) under rainfed **(A, C)** and drought stress **(B, D)** during 2017 **(A, B)** and 2018 **(C, D)**. The contribution of a trait to the principal components is showed by intensity of the color, ranging from the green (lower contribution) to red (higher contribution). Abbreviations: see legend of Table 1.


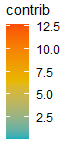

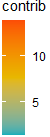


**A**


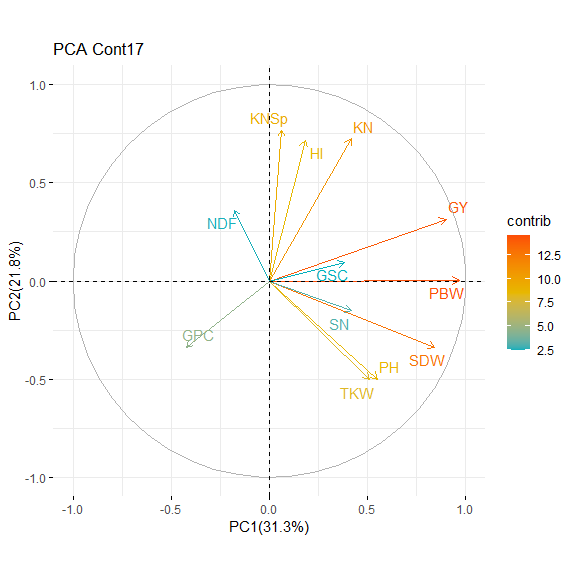

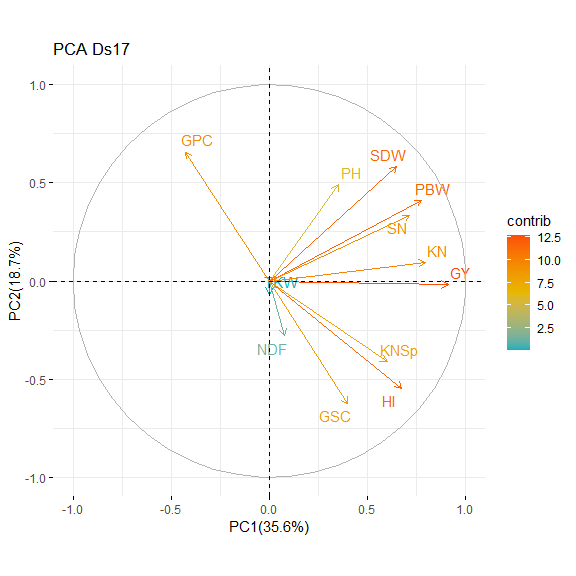


**B**

**C**


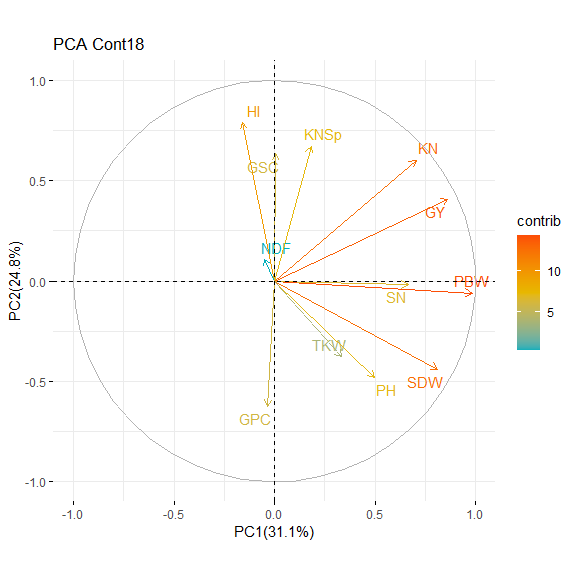


**D**


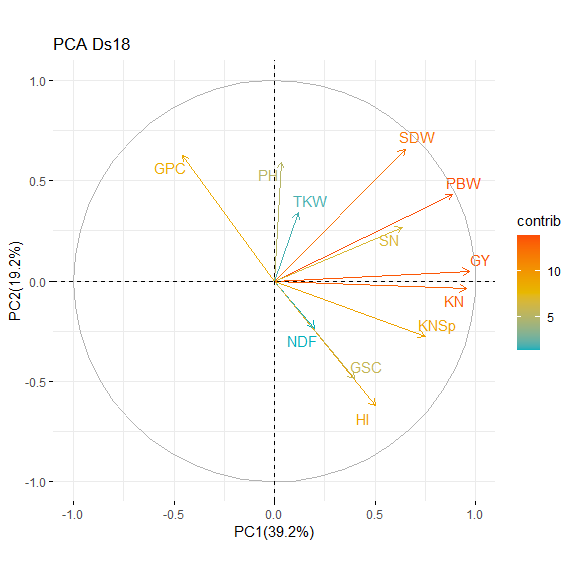


##
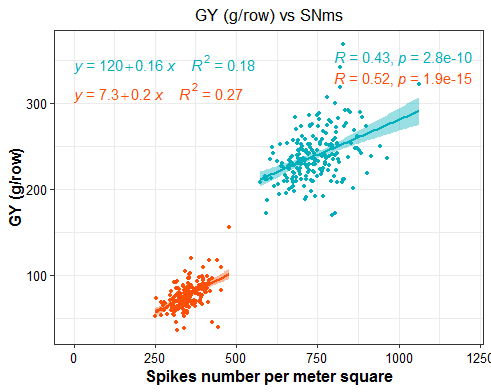

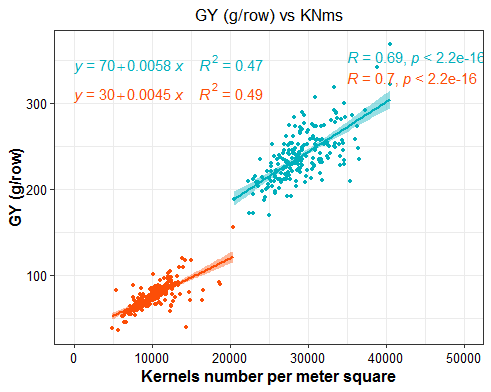

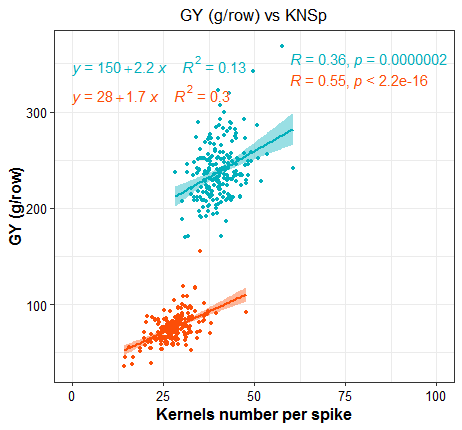

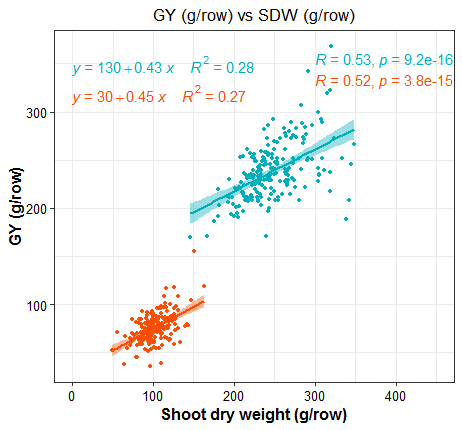
**FIGURE S4 |** Linear regression of GY on yield components traits showing the proportion of the variance in GY explained by the variation in each component trait (R^2^). **(A)** Shoot dry weight; **(B)** Kernels number per spike; **(C)** Kernels number per meter square; **(D)** Spike number per meter square; **(E)** Thousand kernels weight.

**D**

**C**

**E**

**B**

**A**


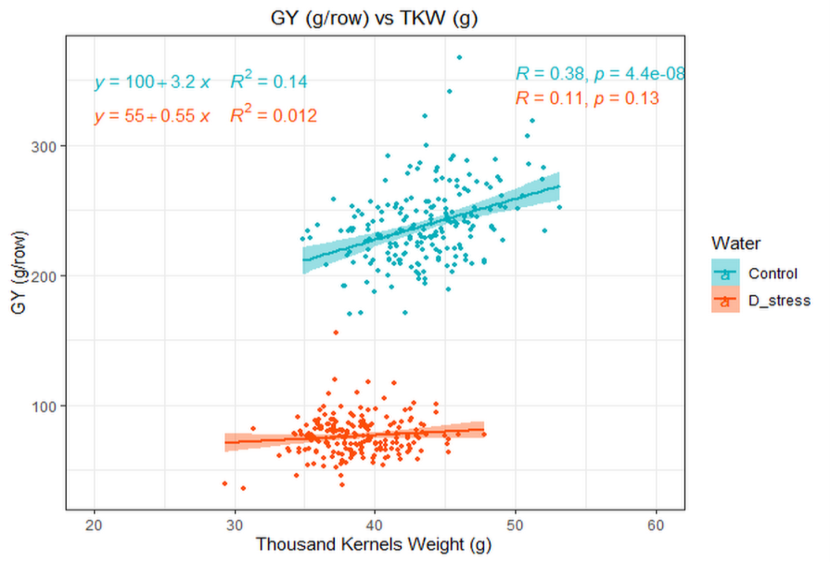

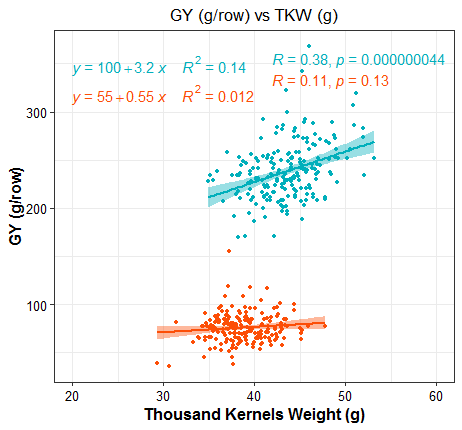


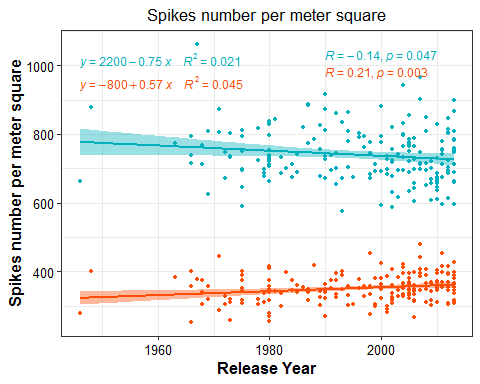
**
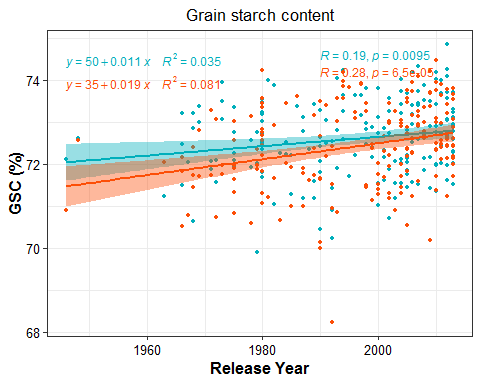

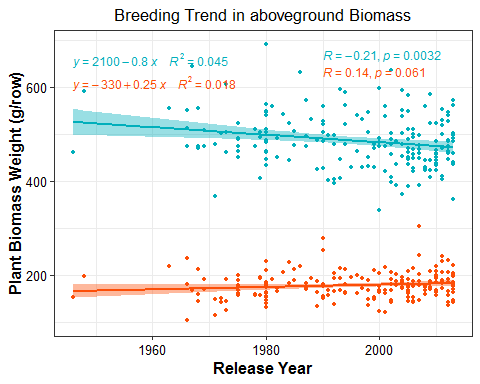

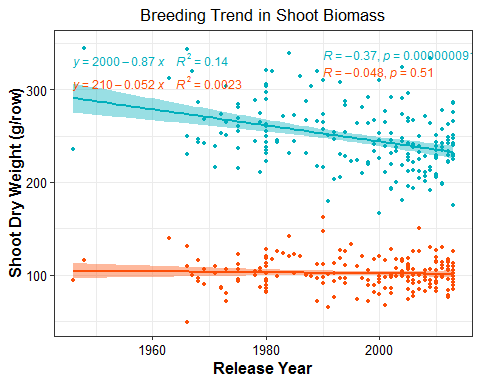

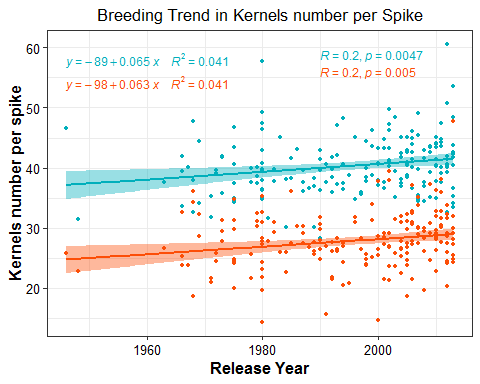

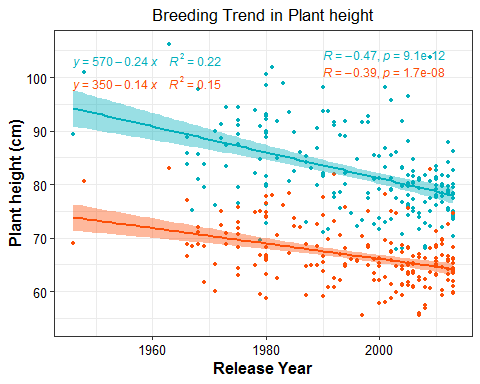

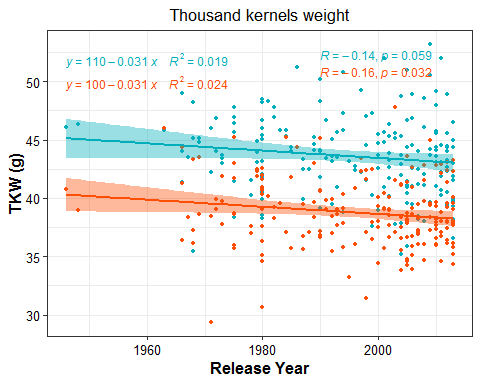

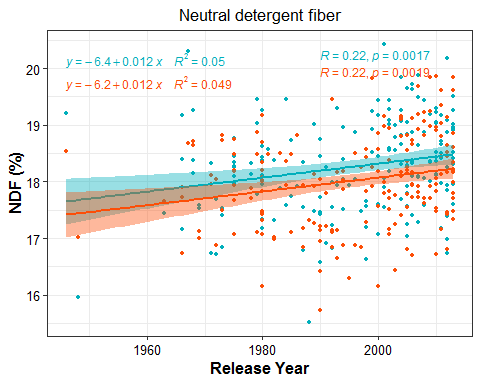

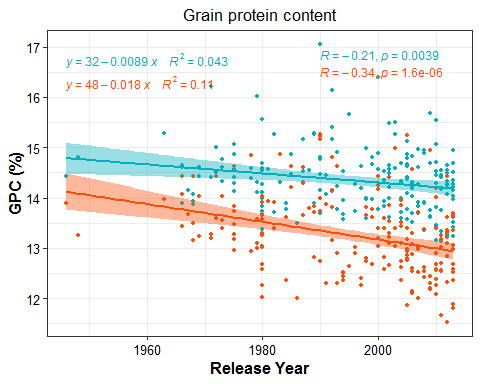
FIGURE S5 |** Regression plots showing breeding progress in agronomic traits and grain quality on Blues values for two growing seasons. Each dot represents a blue value of a cultivars and the colored area represents the confidence interval of the regression line. The slopes of the linear regression lines (green lines for rainfed condition and orange values for droughts stress field) are referred to absolute breeding progress and the relative breeding progress is the ratio between the values in 2010 and 1980 as show in Table 1.


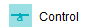

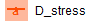

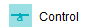

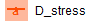

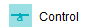

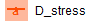


**
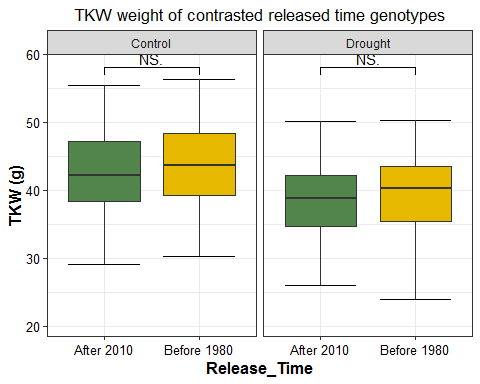

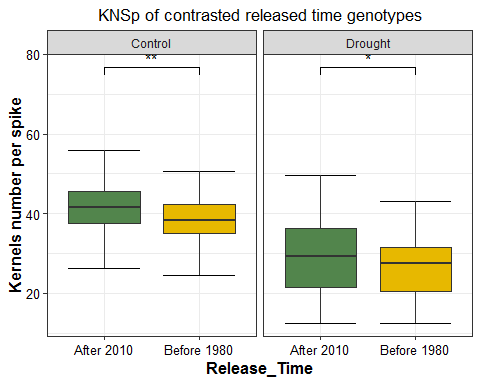
**
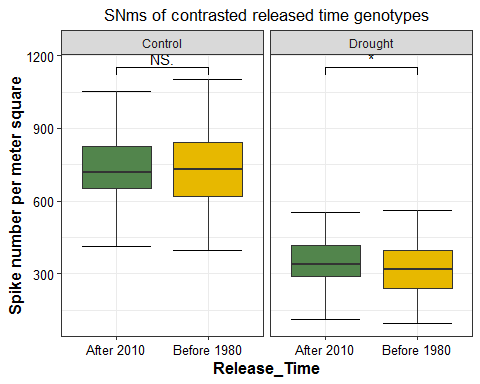
**
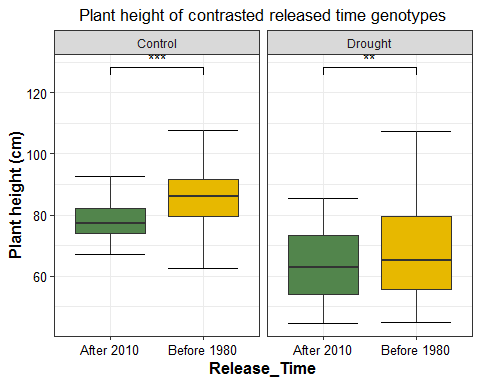

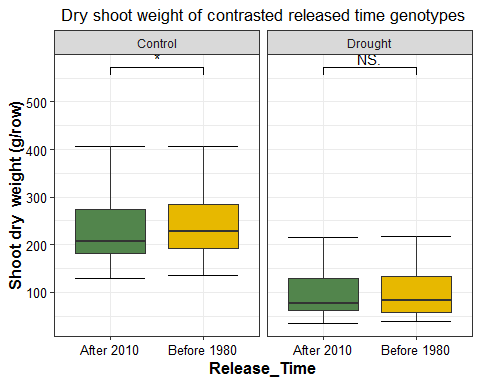

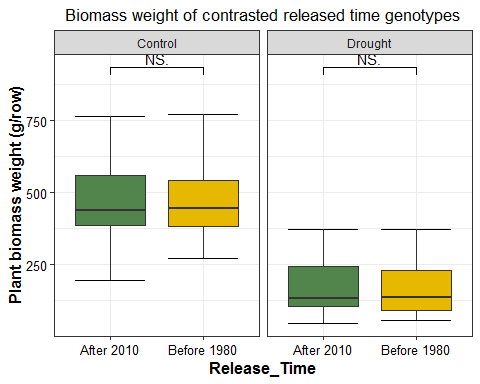

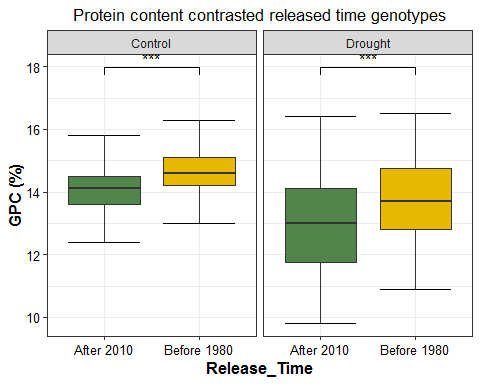

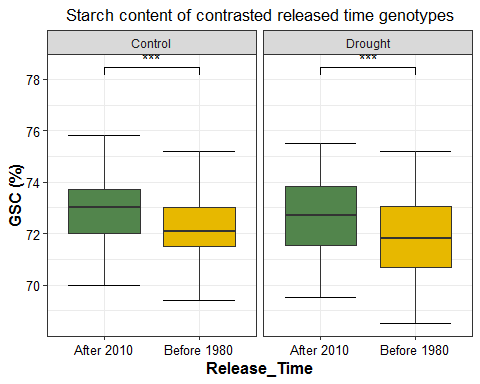

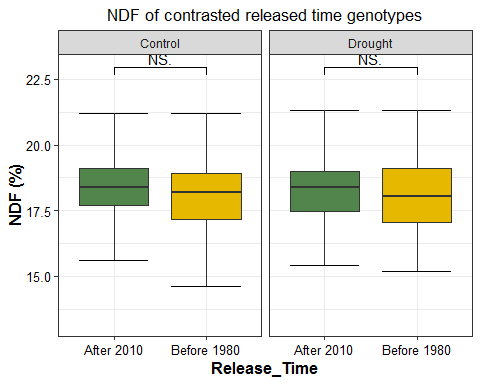
FIGURE S6 |** Comparison of breeding progress in agronomic and grain quality traits between two contrasting years of release groups under control and drought stress conditions. The oldest genotypes were released before 1980 while the newest were released after 2010.

## **FIGURE S7 |** Number of SNP-clusters with defined number of MTAs, illustrating the SNP-clusters having only one MTAs to SNP-clusters with 20MTAs

## **FIGURE S8 |** Circular Manhattan plots displaying association mapping for GSC. **(A)** GSC under both conditions in 2017; **(B)** GSC under both conditions in 2018; **(C)** GSC mean under drought and rainfed; **(D)** GSC overall mean. The dotted square highlighted significant (P<10^-3^ in green color and P<10^-4^ in red color) and consistent MTAs were detected on chromosomes 3A and 5D


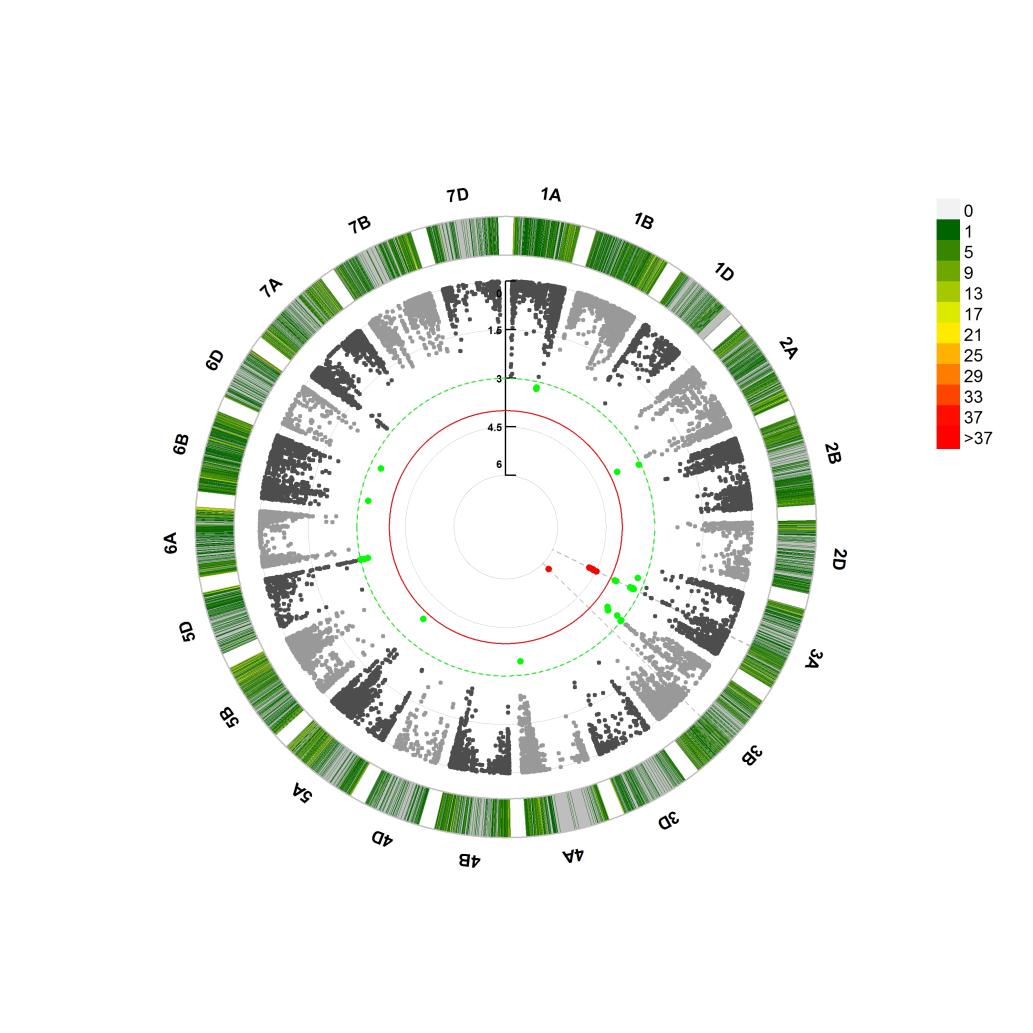


**D**


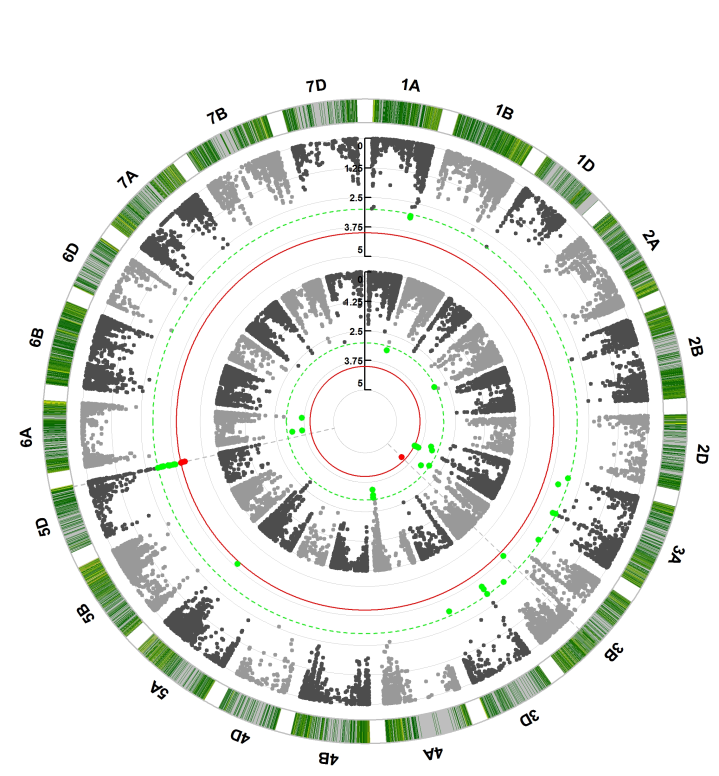


**C**


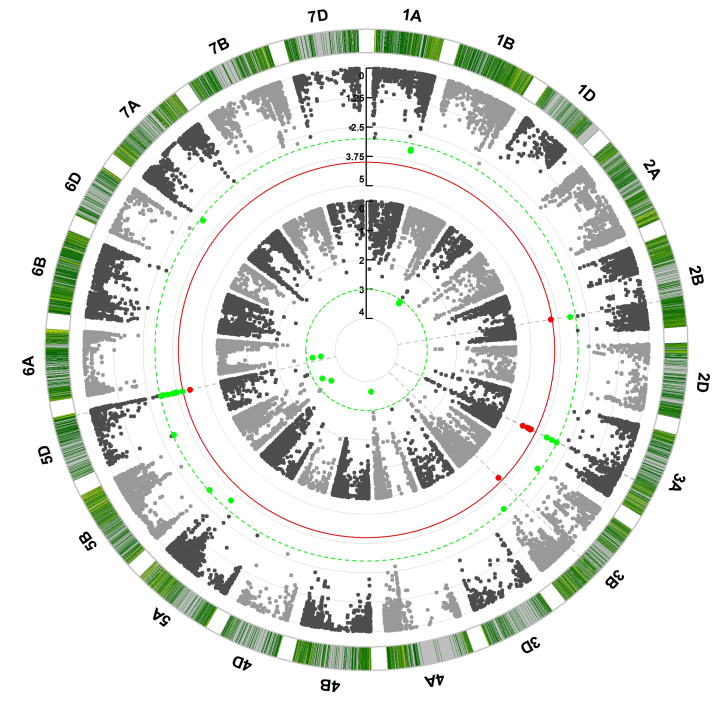


**A**


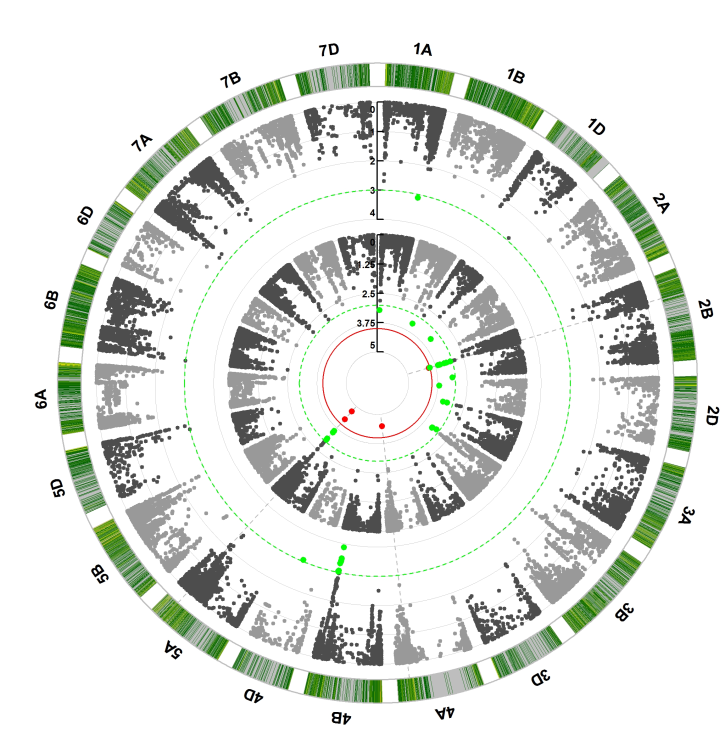


**B**

## **FIGURE S9 |** Effect of AX-158576764 haplotype bloc of chromosome 3A on **(A)** GSC and **(B)** GY under drought stress conditions; **(C)** GSC and **(D)** GY under rainfed conditions.


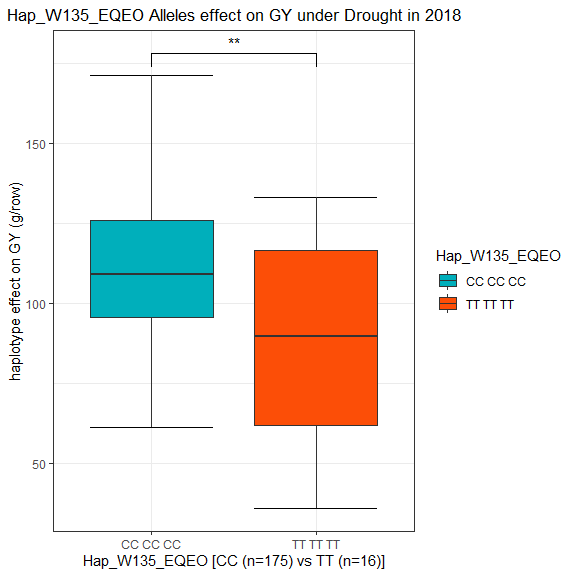


Haplo-bloc 3A [CC (n=175) *vs* TT (n=16)

**B**


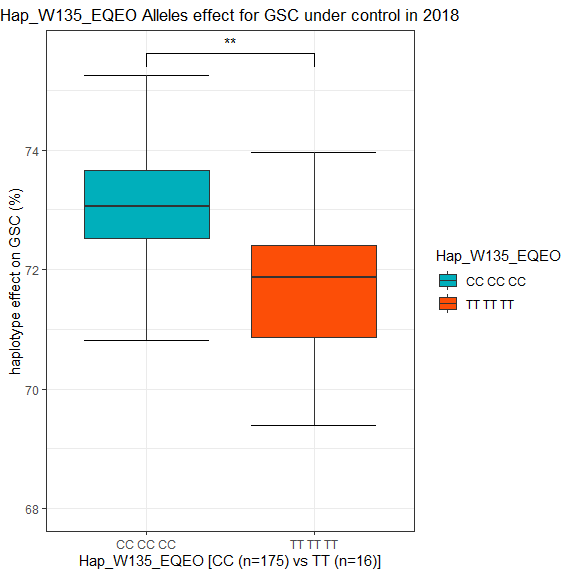


Haplo-bloc 3A [CC (n=175) *vs* TT (n=16)

**C**


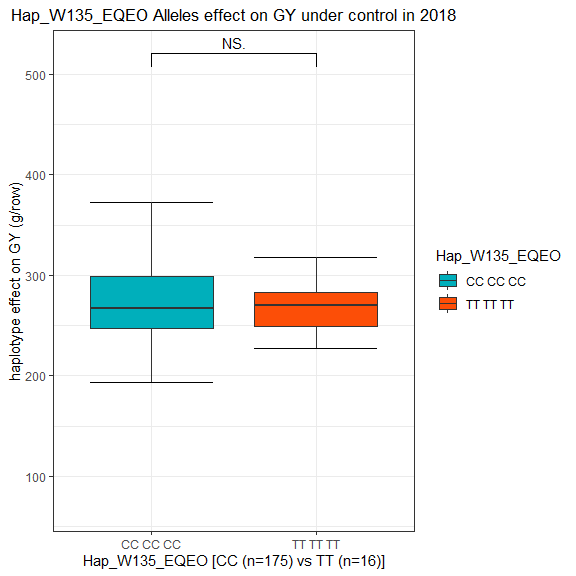


Haplo-bloc 3A [CC (n=175) *vs* TT (n=16)

**D**


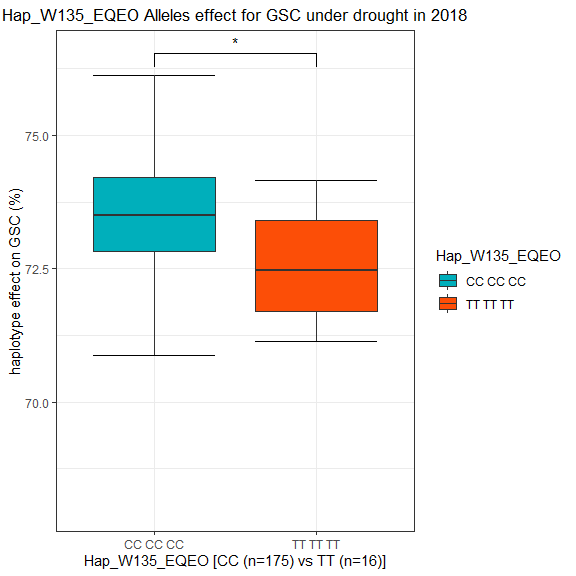


Haplo-bloc 3A [CC (n=175) *vs* TT (n=16)

**A**
